# Hololectin Interdomain Linker Determines Asparaginyl Endopeptidase-Mediated Maturation of Antifungal Hevein-Like Peptides in Oats

**DOI:** 10.3389/fpls.2022.899740

**Published:** 2022-05-10

**Authors:** Shining Loo, Stephanie V. Tay, Antony Kam, Warren Lee, James P. Tam

**Affiliations:** School of Biological Sciences, Nanyang Technological University, Singapore, Singapore

**Keywords:** hololectin, hevein, oats, biosynthesis, celiac diseases, anti-fungal, asparaginyl endopeptidase

## Abstract

Heveins and hevein-containing (hev-) lectins play important roles in stress and pathogenic responses in plants but cause health concerns in humans. Hev-hololectins contain multiple modular hev-peptide domains and are abundantly present in cereals and pseudocereals. However, it is unclear why some cereal hev-hololectins are presented as different forms of proteolytically processed proteoforms. Here we show the precursor architectures of hev-hololectins lead to different processing mechanisms to give either hololectins or hevein-like peptides. We used mass spectrometry and datamining to screen hev-peptides from common cereals, and identified from the oat plant *Avena sativa* nine novel hevein-like peptides, avenatide aV1–aV9. Bioinformatic analysis revealed that asparaginyl endopeptidase (AEP) can be responsible for the maturation of the highly homologous avenatides from five oat hev-hololectin precursors, each containing four tandemly repeating, hev-like avenatide domains connected by AEP-susceptible linkers with 13–16 residues in length. Further analysis of cereal hev-hololectins showed that the linker lengths provide a distinguishing feature between their cleavable and non-cleavable precursors, with the cleavables having considerably longer linkers (>13 amino acids) than the non-cleavables (<6 amino acids). A detailed study of avenatide aV1 revealed that it contains eight cysteine residues which form a structurally compact, metabolic-resistant cystine-knotted framework with a well-defined chitin-binding site. Antimicrobial assays showed that avenatide aV1 is anti-fungal and inhibits the growth of phyto-pathogenic fungi. Together, our findings of cleavable and non-cleavable hololectins found in cereals expand our knowledge to their biosynthesis and provide insights for hololectin-related health concerns in human.

## Introduction

Asparaginyl endopeptidases (AEPs), also known as legumains and vacuolar protein endopeptidases (VPEs), are cysteine proteases that cleave the carboxyl-terminal side of Asx (Asp/Asn) (Csoma and Polgár, 1984; Kembhavi et al., 1993; Yamada et al., 2020). Functional AEPs are widely distributed in plants, mammals, protozoan parasites, trematodes like *Schistosoma mansoni*, and insects like ticks, but not in bacteria (Dall and Brandstetter, 2016). In plants, AEPs play important roles in different plant organs and different stages of plant development and death. They are involved in the processing of peptides, proteins and their precursors like seed storage proteins, for growth and development as well as regulating programmed cell death and environmental stress responses (Vorster et al., 2019). In animals, AEPs are pivotal in the endosome/lysosomal degradation system and are implicated in antigen processing (Manoury et al., 1998, 2002).

Asparaginyl endopeptidases (C13 family) share the cysteine and histidine residues in the active site with other cysteine proteases such as papains (C1 family) and caspases (C14 family), but have little sequence similarity to them (Rawlings et al., 2010). Functionally, AEPs can mediate the biosynthesis of peptides and proteins through selective proteolysis of exposed Asx residues in the mature domains. Recent reports include the maturation of cysteine-rich peptides with protease inhibitory and insecticidal activities, such as roseltides from *Hibiscus sabdariffa* and jasmintides from *Jasminum sambac* (Loo et al., 2016; Kumari et al., 2018; Kam et al., 2019a,b). Unlike other cysteine proteases which solely break peptide bonds, certain AEPs can reverse their enzymatic direction to act as ligases to form peptide bonds (Nguyen et al., 2014, 2015; Harris et al., 2015; Hemu et al., 2019; Du et al., 2020; Chen et al., 2021; Liew et al., 2021). This highly unusual ligating function assisted the AEP-mediated biosynthesis of cyclic peptides through head-to-tail cyclization, forming cyclotides, sunflower seed trypsin inhibitors, and orbitides from seed storage proteins (Mylne et al., 2011; Fisher et al., 2018; Franke et al., 2018). The dual function of AEPs further enable them to act as a splicing enzyme, through a sequence of post-translational cut-and-join events, in the maturation of the cyclic trypsin inhibitor MCoTI-I/II from *Momordica cochinchinese* of the squash family, and circular-permutated plant lectins (Carrington et al., 1985; Bowles et al., 1986; Mylne et al., 2012; Du et al., 2020; Liew et al., 2021; Nonis et al., 2021).

Plant lectins are a superfamily of carbohydrate-binding proteins that serve as defense mechanisms against other plants and fungi (Peumans and Van Damme, 1995; Van Damme et al., 2008; Tsaneva and Van Damme, 2020). They are classified based on the number of carbohydrate-binding domains present in their mature sequences (Peumans and Van Damme, 1995; Van Damme et al., 2008). An example is the chitin-binding domain which interacts with chitin, a common naturally-occurring polysaccharide found in the exoskeleton of insects and the cell wall of fungi (Lenardon et al., 2010). Merolectins, chimerolectins, and hololectins represent three main types of plant lectins containing chitin-binding domains (Peumans et al., 2001; Porto et al., 2012).

Merolectins, the simplest and smallest form of lectins, have a single carbohydrate-binding domain of 29-46 amino acid residues (Peumans et al., 2001). A representative example of merolectin is hevein (hev), a cysteine-rich peptide (CRP) which is derived from the rubber tree (*Hevea brasiliensis*) and was the first reported chitin-binding peptide (Archer, 1960; Rodriguez-Romero et al., 1991; Van Parijs et al., 1991; Andersen et al., 1993; Gidrol et al., 1994). Chimerolectins, such as the *Urtica dioica* agglutinin (UDA), are chimeras which have singly- or tandemly-arrayed hev-peptide domains with a protein cargo such as chitinases (Does et al., 1999; Peumans et al., 2001). In contrast, hololectins have tandem repeats of hev-peptide domains (2–7 repeats), but lack a protein cargo (Peumans et al., 2001).

Heveins, hev-like peptides, and hev-peptide domains are characterized by a conserved cysteine motif, CXnCXnCCXnCXnC which possesses a tandemly connecting CC motif at CysIII and CysIV, and a cystine-knot disulfide connectivity (Rodriguez-Romero et al., 1991; Andersen et al., 1993). The chitin-binding site of heveins consists of a SXφGφ motif in intercystine loop 3, and in loop 4, a GXXXXφ motif (X represents any amino acid and φ represents aromatic acid residues, Phe, Tyr or Trp) (Tam et al., 2015). The conserved chitin-binding site (accession no. PS00026) recognizes and binds to planar chitin monomers (Kini et al., 2015, 2017).

Common cereals such as wheat, rye, barley, oats, corn, and rice contain a high concentration of hololectins (De Punder and Pruimboom, 2013). Representative examples include wheat germ agglutinin (WGA) from wheat, oryza sativa agglutinin (OSA) from rice, and barley hololectins (Wright et al., 1985; Lerner and Raikhel, 1989; Smith and Raikhel, 1989; Zhang et al., 2000). WGA, a 200-amino-acid protein, is present at ∼0.5 g/kg in wheat germ (Peumans and Van Damme, 1996) and contains four interconnected hev-peptide domains (Wright et al., 1985; Smith and Raikhel, 1989). The multivalent hev-peptide domains in WGA and other hololectins play important roles in their cell agglutination activities (Mishra et al., 2019). In addition, the avidity contributed by the repeating hev-peptide domains in WGA that binds strongly not only to chitin found in fungi, but also sialic acid found in the gastrointestinal tract, could trigger celiac diseases (Shaw et al., 1991; De Punder and Pruimboom, 2013). Thus, the presence of hololectins in edible cereal might be a health concern (De Punder and Pruimboom, 2013).

In general, cereal hololectins are processed and released as a protein with multiple hev-peptide domains from their respective precursors without further bioprocessing by an endopeptidase during their maturation (Peumans et al., 2001). Recently, our laboratory identified a cleavable hololectin from *Chenopodium quinoa*, a common edible pseudocereal (Loo et al., 2021b). The quinoa hololectin precursor which contains two hev-peptide domains is processed by a cathepsin-like endopeptidase to release hev-like chenotides, which are anti-fungal (Loo et al., 2021b). Thus, unlike most hololectin precursors which are resistant to proteolytic processing during their maturation, the quinoa hololectin precursors are cleaved to give two identical hev-like peptides. Apart from chenotides, the only other known example of cleavable-hololectins is Sm-Amp-1 from *Stellaria media* (Slavokhotova et al., 2017). However, the molecular basis underpinning the difference between cleavable and non-cleavable hololectins in their biosynthesis remains undetermined.

Here, we report the identification of a novel family of anti-fungal hev-like peptides from oats (*Avena sativa*) termed avenatides aV1–aV9 which are derived from hololectin precursors. Unlike majority of cereal hololectins which are presented as proteoforms with multiple hev-domains, oat hololectins are presented as proteolytically processed proteoforms with a single hev-domain. A structural feature distinguishing these two families can be found in their precursor architecture and interdomain linkers that contribute to their different mechanisms of biosynthesis.

## Materials and Methods

### Plant Material

All plant materials were purchased from local grocery stores, including *Avena sativa, Briza maxima, Cajanus cajan, Coix lacryma, Elymus canadensis, Glycine max, Phaseolus vulgaris, Hordeum jubatum, Hordeum vulgare, Secale cereale, Sorghum bicolor, Triticum aestivum, Vigna umbellate, Vigna radiata, Vigna unguiculata*, and *Vigna angularis*. Authentications were done by Mr. Paul Leong from the Singapore Botany Center based on macroscopic and microscopic analyses. Voucher samples were deposited at the Nanyang Technological University Herbarium, School of Biological Sciences, Singapore.

### Isolation and Purification of Avenatide aV1

Small scale screening of oats and other cereals and legumes were performed by vortexing 0.1 g oats with 1 mL water for 1 h. The crude extract was centrifuged at 9,500 rpm for 10 min and the resulting supernatant was subjected to a C18 Zip-tip and eluted with 80% ACN.

For large scale extraction, 2 kg of oats were homogenized in 20 L of water for 3 h. The crude extracts were centrifuged at 9,500 rpm for 20 min at 4°C. The supernatant was filtered before loading on a flash column packed with 500 g C18 powder (Grace, MD, United States) in a Büchner funnel. Elution was performed using increasing concentrations of ethanol (20–80%). Eluents containing avenatide aV1 were pooled and purified using multiple rounds of SCX- and RP-HPLC in which fractions from SCX-HPLC containing avenatide aV1 were pooled and further purified by RP-HPLC. MALDI-TOF MS was used to identify the presence and assess the purity of avenatide aV1 in the eluted fractions.

### Sequence Determination

The primary sequences of avenatides were determined by LC-MS/MS sequencing as described previously (Loo et al., 2016). The RP-HPLC-enriched avenatide samples were re-dissolved in 20 mM DTT at 37°C for 1 h followed by *S*-alkylation with 200 mM iodoacetamide at 37°C for 1 h. The mixture was desalted with a C18 Zip-tip and subjected to analysis on a Dionex UltiMate 3000 UHPLC system equipped with an Orbitrap Elite mass spectrometer (Thermo Fisher Scientific Inc., Bremen, Germany). The composition of mobile phase A and B were 0.1% FA in deionized water and 0.1% FA in 90% acetonitrile with 10% deionized water, respectively. Mass spectra were acquired with LTQ Tune Plus software (Thermo Fisher Scientific, Bremen, Germany) using a positive mode with alternating Full FT-MS as previously described (Kumari et al., 2018). The data analysis were performed using PEAKS studio (version 7.519, Bioinformatics Solutions, Waterloo, ON, Canada) with a precursor ion tolerance of 10 ppm and fragment ion tolerance of 0.05 Da. Carbamidomethylation at Cys was set as a fixed modification. Deamidation of Asp and Glu, oxidation of Met, acetylation at Lys and N-term were set as variable modification. Peptide sequencing was performed using PEAKS DB protein identification which integrates database search of in-house eight-cysteine hev-peptide library with *de novo* sequencing using the following filtering parameters: peptide hit threshold (-10logP) was set as 30.0 and *de novo* score (%) threshold was set as 15. The data generated in this study are publicly available via the ProteomeXchange consortium through the partner repository JPOST (Okuda et al., 2017). JPOST accession: PXD033161.

### Nuclear Magnetic Resonance Structural Study

All nuclear magnetic resonance (NMR) experiments were conducted on a BRUKER Avance 800 NMR spectrometer with a cryogenic probe at 25°C. The concentration of each peptide was around 1 mM, in a solution containing 5% D_2_O and 95% H_2_O (pH 3.5). For 1H, 1H-2D TOCSY and NOESY, the mixing times were 80 and 200 ms, respectively. The spectrum width was 12 ppm for both dimensions. The NMR spectra were processed using NMRPipe software (Delaglio et al., 1995). All data analysis were performed using Sparky software based on results of the 2D NOESY and TOCSY experiment. The proton chemical shift assignments for each amino acid residue were achieved by 2D TOCSY and NOESY while the proton-proton distances restraints were obtained from 2D NOESY based on the intensities of NOE cross-peaks. The solution structures of avenatide aV1 were calculated using CNSsolve 1.3 software. Proton-proton distance restraints and hydrogen bonds were employed in a standard simulated annealing protocol. The distance restraints were divided into three classes based on NOE cross-peak intensities: strong, 1.8 < d < 2.9 Å, medium, 1.8 < d < 3.5 Å and weak, 1.8 A < d < 5 Å. Eight hydrogen bonds were used in the structure calculation. A total of 100 structures were calculated and the 10 lowest energy structures were chosen for data statistics and presentation. The structure was verified using the PROCHECK program (Laskowski et al., 1996) and presented using Chimera version 1.6.2 (Huang et al., 1996; Pettersen et al., 2004). Accession code(s): PDB ID 6M5C.

### Chitin-Binding Assay

A chitin-binding assay was performed as described previously (Loo et al., 2021b). Briefly, *S*-alkylated and purified avenatide aV1 were mixed with chitin beads (80 μL) (New England BioLabs, United Kingdom) in chitin binding buffer and incubated at 25°C for 30 min. After incubation, the mixture was washed with chitin binding buffer to remove unbound compounds. Elution of bound peptide was performed with 1 M acetic acid. The supernatant and eluent were analyzed using RP-UPLC and MALDI-TOF MS to assess binding and elution.

### Peptide Stability Assay

Purified avenatides aV1 and *S*-alkylated aV1 were incubated under the stated conditions and recommended buffer solution. At each time interval, aliquots of samples were taken and RP-UPLC was performed in triplicate.

### Exoproteolytic Enzyme Stability Assay

Purified avenatide aV1 (200 μM) were added to 50 mM Tris-HCl, 100 mM sodium chloride with 100 nM carboxypeptidase A or 20 mM tricine and 0.05% bovine serum albumin (pH 8.0) with 20 U/mL aminopeptidase I. The mixture was incubated in a 37°C water bath for 4 h. At each time-point (0 and 6 h), 20 μL of the treated sample was aliquoted and quenched with 5 μL 1 M hydrochloric acid. RP-UPLC was performed to determine the amounts of avenatide aV1 present before and after treatment.

### Anti-fungal Assay

Four phyto-pathogenic fungal strains from the China Center of Industrial Culture Collection (Beijing, China) were used to examine the anti-fungal activity of avenatide aV1: *Alternaria alternata* (CICC 2465), *Curvularia lunata* (CICC 40301), *Fusarium oxysporum* (CICC 2532), and *Rhizoctonia solani* (CICC 40259). Fungal strains were grown on potato dextrose agar plates at 25°C.

The half maximal inhibitory concentration levels (IC_50_) of avenatide aV1 were determined using a microbroth dilution assay (Loo et al., 2021b). Fungal spores were harvested from a 4-day old, actively growing fungal plate and suspended in half-strength potato dextrose broth. In the 96-well microplate, 1 × 10^5^ cells/mL of spore suspension was mixed with peptides at varying concentrations and incubated at 25°C for 24 h. The cells were then fixed with 100% methanol for 15 min. Staining was done for 45 min with crystal violet dye. MilliQ water was used to remove excess dye. Elution was performed using 1:1 (v/v) ethanol/0.1 N HCl. Absorbance was measured at 570 nm.

### Data-Mining and Bioinformatics Analysis

Genes encoding avenatides, hololectin OAT_OCH1-5 (accession number: GO581539.1, GO581912.1, GO582252.1, GO583188.1, GO585827.1) were obtained from the NCBI GenBank and translated using the ExPaSy translation tool. Signal peptide cleavage sites were identified using SignalP 4.0 (Petersen et al., 2011).

Data-mining was performed to collect datasets for hev-peptide domain precursor sequences using a combination of motif searches and BLAST algorithms. For motif searches, the “Search Sequence Database” function in MOTIF Search^1^ was used to interrogate Genbank, UniProt and RefSeq databases using a common hev-peptide domain input; C-{C}n-C-{C}n-C-C-{C}n-C-{C}n-C-{C}n-C-{C}n-C, where “{C}” represents any amino acid except cysteine. For data-mining using BLAST, avenatide precursor sequences were used to perform BLAST on NCBI Genbank and OneKP. BLAST searches were confined to the taxa “Viridiplantae” (taxid:33090). Full sequences were retrieved from the databases and converted to.fasta formats. Then, sequences were re-interrogated using the “Search Motif Library” function with the Pfam motif library selected in MOTIF Search^2^ to identify sequences with chitin-binding domains. Sequences were then sorted to identify hololectins containing multiple chitin-binding domains. Bioinformatics analysis was performed by identification of hololectin linkers which are defined as the sequences between two cysteinyl residues of two adjacent hev-peptide domains.

## Results

### Mass Spectrometry Profiling of Hev-Peptides From Oats, Cereals and Legumes

We used a mass spectrometry (MS)-driven approach to profile hev-like peptides in the aqueous extract of oats, and selected cereals and legumes, including *B. maxima*, *C. cajan*, *C. lacryma*, *E. canadensis*, *G. max*, *P. vulgaris*, *H. jubatum*, *H. vulgare*, *S. cereale*, *S. bicolor*, *T. aestivum*, *V. umbellate*, *V. radiata*, *V. unguiculata*, and *V. angularis* (Loo et al., 2016, 2017, 2021a,b; Tam et al., 2018; Kam et al., 2019a,b; Supplementary Figures 1–19). Since hev-peptides contain 38–42 amino acids and eight cysteine residues, we focused on peaks around 4 kDa in their MS profiles. Also, because hev-peptides are CRPs, we performed a mass-shift assay to determine their mass increase, and in turn, their cysteine content. After *S*-reduction of cystine residues with dithiothreitol (DTT) and *S*-alkylation of the liberated cysteine residues with iodoacetamide (IAM), each *S*-alkylated cysteine residue results in an increase of 58 Da.

**FIGURE 1:**
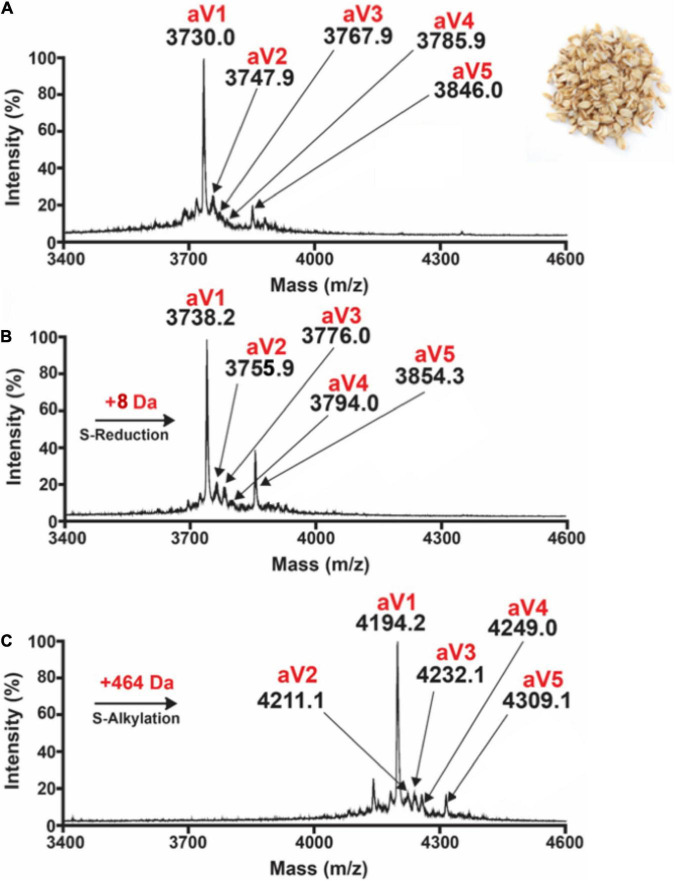
MALDI-TOF MS profile of **(A)** aqueous oat extracts. Clusters of peaks between 2,000 and 4,000 Da indicate the presence of putative cysteine-rich peptides. Avenatides aV1–aV5 are labeled at corresponding peaks. **(B)** MALDI-TOF MS profile of aqueous oat extracts after *S*-reduction by DTT, and **(C)** *S*-alkylation by iodoacetamide (IAM) to give their corresponding linear forms and a gain of 58 Da for each *S*-alkylated Cys. Based on the increase of 464 Da, each avenatide is calculated to contain 8 cysteine residues.

Preliminary profiling by MALDI-TOF-MS on 20 crop samples revealed the absence of prominent peaks around 4 kDa with eight cysteine residues in the aqueous extracts of *B. maxima*, *C. cajan*, *C. lacryma*, *E. canadensis*, *G. max*, *P. vulgaris*, *H. jubatum*, *H. vulgare*, *S. cereale*, *S. bicolor*, *T. aestivum*, *V. umbellate*, *V. radiata*, *V. unguiculata*, and *V. angularis*. In contrast, oats displayed prominent clusters of putative CRPs around 4,000 Da (Figure 1A and Supplementary Figures 1–19). From these clusters, five avenatides, termed aV1-aV5, with relative monoisotopic molecular weights [M + H]^+^ of 3,730, 3,747, 3,767, 3,785, 3,846, and 3,858 Da, respectively, were identified and annotated. Mass-shift assay revealed a mass increase of 464 Da, indicating that avenatide aV1–aV5 are putative eight-Cys hev-peptides (Figures 1B,C).

### Primary Sequence of Avenatides

**FIGURE 2:**
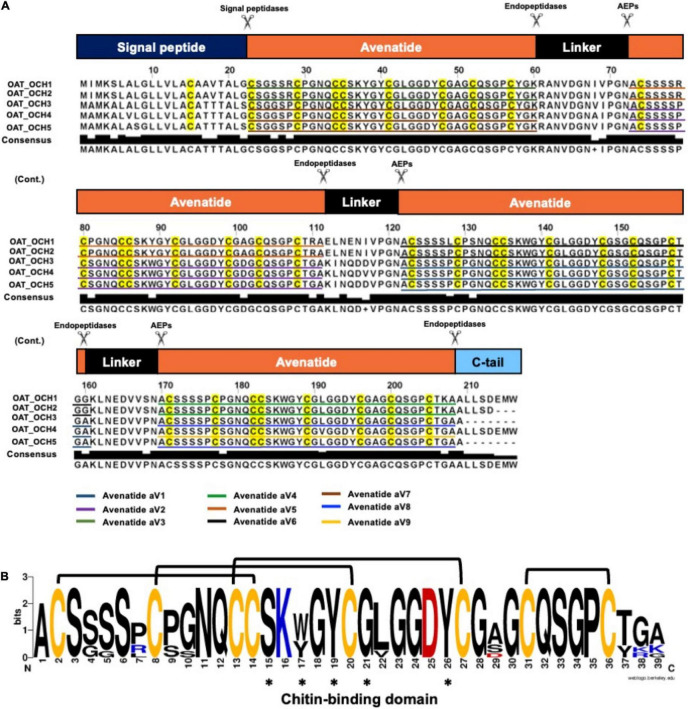
Biosynthetic precursors and sequence comparison of avenatides. **(A)** Alignment of avenatide precursor sequences. Avenatide precursors contain an N-terminal signal peptide, four tandem repeating mature hev-peptide domains, and a C-terminal tail. The signal peptide is cleaved by signal peptidase, whereas the C-terminal domain and linkers are likely cleaved by an asparaginyl endopeptidase at the conserved Asn-Ala dipeptide motif to release the mature avenatide domain with Ala as the N-terminal amino acid in avenatides. **(B)** Consensus sequence of avenatides. The disulfide connectivity is based on the NMR-determined structure shown in Figure 3 and amino acid residues contributing to the conserved chitin-binding sites are indicated by asterisks.

To investigate the identities of avenatides, we used LC-MS/MS *de novo* sequencing assisted by database search with in-house hev-peptide library for determining the primary sequence of avenatides aV1-aV5 from oat extracts. This combined approach yielded the primary sequence of avenatide aV1 as A**C**SSSSP**C**PGNQ**CC**SKWGY**C**GLGGDY**C**GSG**C**QSGP**C**TGA. In addition to avenatides aV1-aV5 (Supplementary Figures 20, 21), a database search from NCBI revealed the primary sequences of avenatides aV6–aV9 (Figure 2). The primary sequences of avenatides aV1–aV9 have 38–39 residues and are highly homologous (>90% similarity), with an overall charge ranging from -1 to +2. Similar to other hev-peptides and hev-peptide domains, all avenatides are both Cys- and Gly-rich (aV1 contains 9 Gly) and contain an evolutionarily conserved hev-peptide-like cysteine motif (CX_n_CX_n_CCX_n_CX_n_C) with a tandemly-connecting cysteine at the third and fourth position, and a chitin-binding site characterized by a highly conserved motif SK**X**(Y/W)G**Y** in intercysteine loop 3 (Table 1), followed by a GLGGD**Y** motif in loop 4 (the three invariable aromatic amino acids lettered in bold). Examples of other 8-Cys hev-peptides with similar cysteine motif and chitin binding site include ginkgotide gB1 from *Ginkgo biloba* and morintide mO1 from *Moringa olfeia* (Wong et al., 2016; Kini et al., 2017). In addition, the primary sequence of avenatides showed ∼70% similarity to avesin A, the first and only oat-derived hev-peptide domain, which was reported in 2003 (Li and Claeson, 2003). Avesin A is a 37-residue hev-peptide domain that has the primary sequence of WSG**C**SP**C**PGNE**CC**SKYGY**C**GLGGDY**C**GAG**C**QSGP**C**YG, and an overall -1 charge (Li and Claeson, 2003).

**TABLE 1:** Sequence comparison of the mature peptide sequences of avenatides and reported eight-cystine-hevein-like peptides.

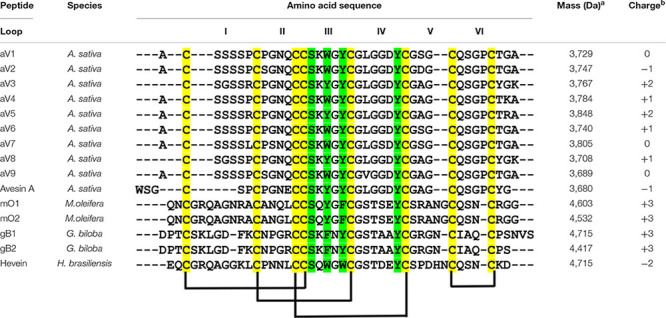

*^a^Mass (Da): represents the experimentally found molecular weight.*

*^b^Charge: represents the total charge of the molecule, and calculated by the sum of positive (lysine, arginine, and histidine residues) and negative (glutamate and aspartate residues) charges.*

*Colored in green represents chitin binding domain. Colored in yellow represents cysteine motif.*

### Biosynthesis and Precursor Architecture of Avenatides

From the NCBI database, we identified five full-length avenatide precursors, which we termed oat-cleavable hololectins (OCH1-5). Figure 2A shows the avenatide-precursors OCH1-5 as a three-domain hololectin precursor consisted of: (1) an N-terminal signal peptide, (2) four tandem-repeating mature domains of chitin-binding-avenatides joined by three linkers, and (3) a C-terminal tail. Sequence comparison showed that the tandem domains of OCH1 and OCH2 are identical and consisted of avenatides aV5, aV6, and aV4, with linkers between each avenatide domain. Similar architectural arrangements were found in OCH3-5 which consisted of avenatides aV7, aV2, aV1, aV8, and aV9. The linkers in avenatide precursors OCH1-5, also known as hinges or connecting peptides between two different avenatide domains, have 13–16 residues and are Asn/Asp-rich (> 20%). The presence of cleavage sites located at the N-terminal Asn-Ala residues of each avenatide domain suggests the involvement of asparaginyl endopeptidases in their bioprocessing to give avenatides aV1-9 with Ala as the N-terminus. Indeed, the predicted and calculated masses from the mass spectrometry of avenatides aV1, aV2, aV4, and aV5, further supported the AEP-mediated cleavage at the Asn-Ala dipeptide site. This finding suggested that the hydrolase activity of AEP could be involved in the release of each avenatide.

### Structure of Avenatide aV1 and Biochemical Assay Its Chitin-Binding Activity

**FIGURE 3:**
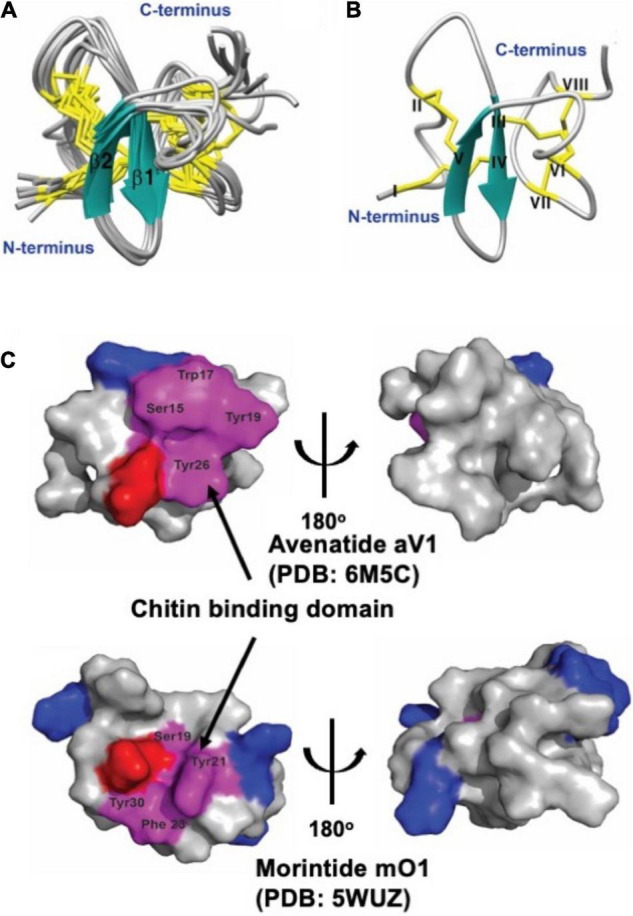
Solution structure of avenatide aV1and comparison of the chitin-binding region with morintide mO1 which belongs to hevein-like domain. **(A)** Superposition of the avenatide aV1 backbone traces from the final 10 ensemble solution structures and restrained energy minimized structure. **(B)** Ribbon representation of avenatide aV1 structure. **(C)** Surface topology comparison of avenatide aV1 (PDB: 6M5C), and morintide mO1 (PDB: 5WUZ). Residues highlighted in purple represent the chitin-binding site. Residues highlighted in blue and red are basic (Arg, His, and Lys) and acidic (Asp, Glu), respectively.

Avenatide aV1-aV9 contain a chitin-binding motif similar to other hev-peptides (Figure 2B). To confirm the disulfide connectivity of avenantide, we used NMR spectroscopy to determine the solution structure of avenatide aV1 (PDB: 6M5C). All spin-spin systems of avenatide aV1 were identified, and approximately 98% of the proton resonances were unambiguously assigned. The solution structure of avenatide aV1 was determined based on a total of 260 NMR-derived distance restraints and eight hydrogen bonds. The NMR ensemble of the 10 lowest-energy avenatide aV1 structures was determined (Figure 3A). The RMSD value of the 10 best structures for residues Ser3-Gly9 and Gly18-Thr37 was 1.23 ± 0.26 Å, and for all heavy atoms was 1.68 ± 0.27 Å (Supplementary Tables 1, 2). The structure of avenatide aV1 was well-defined by several medium- and long-range NOEs consisting of two short extended anti-parallel beta-strands (B1: Cys13-Ser15 and B2: Try19-Gly21) (Figure 3B). Avenatide aV1 has cystine-knot disulfide connectivity at its N terminus and an additional disulfide-bonded loop at its C terminus (CysI–CysIV, CysII–CysV, CysIII–CysVI, CysVII–CysVIII). The N and C termini of avenatide aV1 are not close in proximity. Surface topography of the chitin binding site of avenatide aV1 (Ser-15, Trp-17, Tyr-19 and Tyr-26) revealed its similarity to morintide mO1, a hev-peptides isolated from *Moringa oleifera* (Figure 3C; Kini et al., 2017). Notably, the three conserved aromatic residues, Trp-17, Tyr-19, and Tyr-26 in the avenatide aV1 chitin-binding site, play an essential role in binding to planar chitin monomers (Kini et al., 2015, 2017).

**FIGURE 4:**
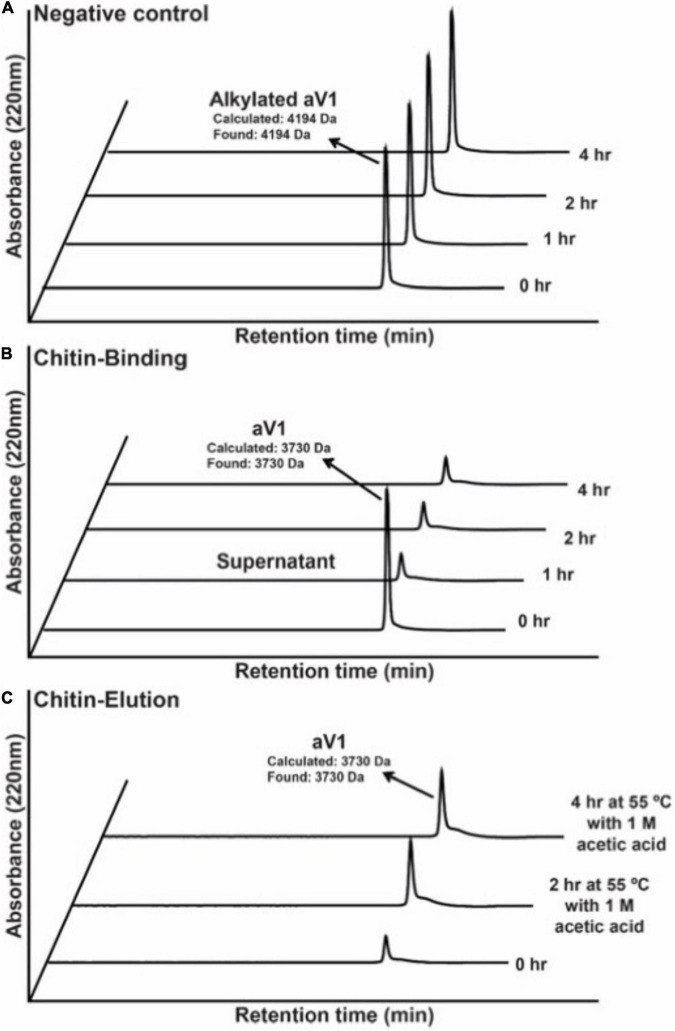
Comparison of chitin-binding activities of **(A)** *S*-alkylated aV1 (iodoacetamido-) and **(B)** avenatide aV1 (iodoacetamido-) using chitin resins. The supernatants were analyzed by RP-HPLC. **(C)** Elution profile of avenatide aV1 from chitin resin using 1M acetic acid at 55°C. The supernatants were analyzed by RP-HPLC.

To confirm the chitin-binding activity of avenatides, the representative avenatide aV1 together with the control, the linear *S*-alkylated aV1, were incubated with chitin beads at 25°C for 1 h. Analysis of the elution profiles by C18 reversed phase high-performance liquid chromatography (RP-HPLC) revealed a complete depletion of avenatide aV1 from the incubation solution, indicating its binding to the chitin beads (Figure 4). Avenatide aV1 binding activity was confirmed by elution with ∼40% 1 M acetic acid at 55°C (Figure 4). In contrast, the linear *S*-alkylated aV1 was not retained by chitin beads, suggesting that the 3-dimensional structure of the aV1 chitin-binding site is important for its chitin-binding activities.

### Avenatide aV1 Is Hyperstable

**FIGURE 5:**
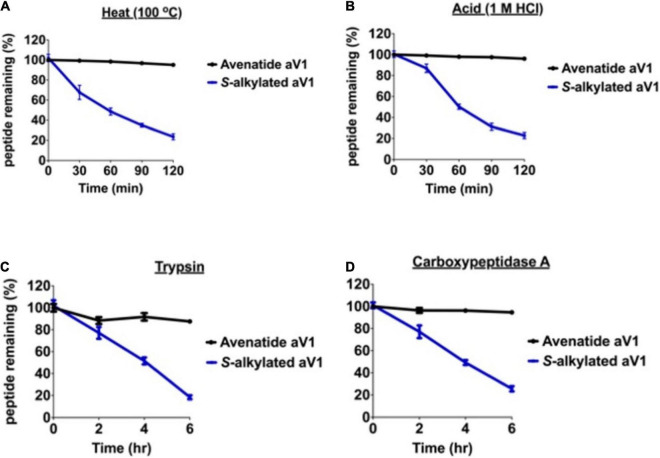
Stability comparison of avenatide aV1 and *S*-alkylated aV1 (iodoacetamido-) under **(A)** heat (95°C), **(B)** acid (1M HCl), **(C)** trypsin, and **(D)** carboxypeptidase A treatment as analyzed by RP-HPLC (*n* = 3).

Cysteine-rich peptides cross-linked by multiple disulfides are known for their stability against heat, acid, and proteolytic degradation (Kini et al., 2015, 2017; Loo et al., 2016, 2017, 2021a,b; Wong et al., 2016; Tam et al., 2018; Kam et al., 2019a,b). To investigate the involvement of the disulfide scaffold on the stability of avenatides, we performed peptide degradation assays on folded avenatide aV1 and compared with its linearized *S*-alkylated form. Our results showed that avenatide aV1 is highly stable against heat-, acid-, endopeptidase- (represented by trypsin), and exopeptidase- (representing by carboxypeptidase A) mediated degradation. In all conditions, >80% of the peptides were retained after treatment, as monitored by RP-HPLC (Figure 5). In contrast, under the same conditions, the linearized *S*-alkylated avenatide aV1 showed substantial reduced stability, indicating the importance of the cystine-knot scaffold in conferring the hyperstability of avenatides.

### Avenatide aV1 Is an Anti-fungal Peptide

**FIGURE 6:**
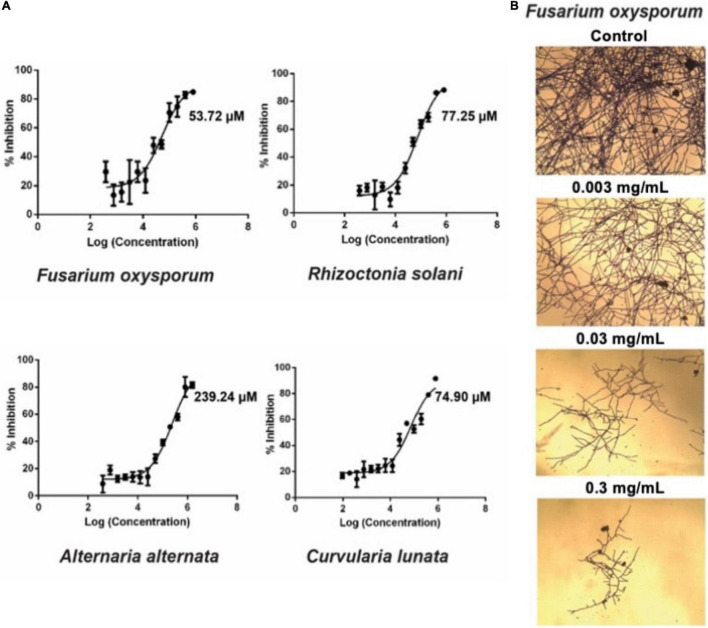
Antifungal assays of avenatide aV1. **(A)** Fungal inhibition of avenatide aV1 against *Fusarium oxysporum*, *Rhizoctonia solani*, *Alternaria alternata*, and *Curvularia lunata*. The IC_50_ was calculated based on the dose-response curve obtained from the micro-broth dilution assay. **(B)** Bright field microscopy of hyphal growth inhibition with avenatide aV1. *F. oxysporum* treated with different concentrations of avenatide aV1. Formation of stunted hyphae ends indicated that avenatide aV1 inhibits hyphal growth at the ends of the fungal mycelia.

To investigate the anti-fungal activities of avenatide aV1, we performed microbroth dilution assay using four phyto-pathogenic fungal strains of *A. alternata*, *C. lunata*, *F. oxysporum*, and *R. solani*. A microbroth dilution assay showed that avenatide aV1 has anti-fungal activity against all four fungal strains as evidenced by IC_50_ values of 239, 74, 53, and 77 μM for *A. alternata*, *C. lunata*, *F. oxysporum*, and *R. solani*, respectively (Figure 6A). To show that avenatide aV1 inhibits hyphae growth, *F. oxysporum* fungal spores were treated with different concentrations of avenatide aV1. Microscopic analysis revealed that avenatide aV1 stunted hyphae growth (Figure 6B).

### Length of Interdomain Linkers in Protein Precursors Determine Cleavable- and Non-cleavable Hololectins in Cereals

To understand the difference between endopeptidase-susceptible and -resistant hololectins that give cleavable- and non-cleavable hololectins, we performed a BLAST search of hololectins containing hev-peptide domains using the NCBI and OneKP database (Johnson et al., 2008; Matasci et al., 2014). The search criteria include the presence of an evolutionarily conserved cysteine motif (CX_n_CX_n_CCX_n_CX_n_C) with a tandemly connecting CC motif at the position of CysIII and CysIV typical of a hev-peptide domain, and a chitin-binding site having a SXXG and GXXXXφ motif at the inter-cysteine loop 3 and 4, respectively (Figure 2). We further refined the search to identify putative AEP-susceptible hololectins based on the presence of Asn/Asp residues in their linkers.

**FIGURE 7:**
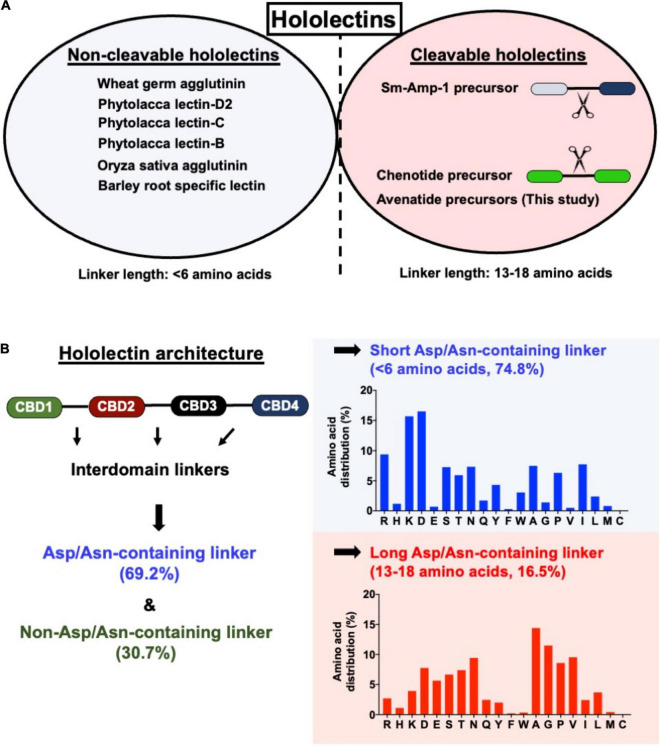
**(A)** Comparison of linker length of reported cleavable hololectins (13–18 amino acids) and non-cleavable hololectins (4–6 amino acids). **(B)** Database search revealed a total of 121 hololectin precursor sequences with 280 hololectin linkers from NCBI and Onekp database. 69.2% are Asn/Asp-containing hololectin linkers that are susceptible to AEP processing. Among these Asn/Asp-containing hololectin linkers, 74.8% are classified as short linkers (<6 amino acids), and 16.5% are long linkers (13–18 amino acids). Short linkers, but not long linkers are rich in positive charge amino acids (e.g., Arg and Lys).

**FIGURE 8:**
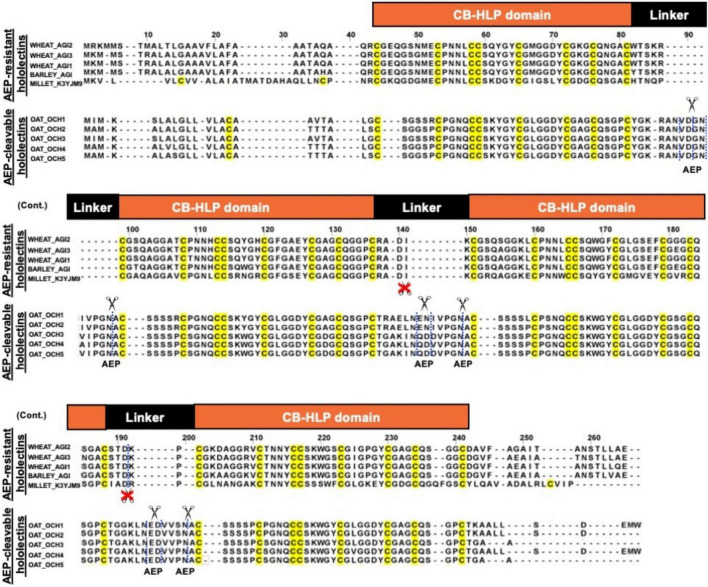
Sequence alignment of AEP-resistant hololectin (e.g., WHEAT_AGI1, P10968; WHEAT_AGI2, P02876; WHEAT_AGI3, XP_037472823.1; BARLEY_AGI, P15312; MILLET_K3YJM9, XP_004972860.1) and putatively AEP-cleavable hololectin OAT_OCH1-5 (e.g., GO581539.1, GO581912.1, GO582252.1, GO583188.1, GO585827.1) precursor sequences from common cereals.

A total of 121 hololectin precursor sequences with 280 hololectin linkers were identified from 44 plant species (Supplementary Figure 22 and Supplementary Tables 3–6). After refinement, 194 putatively AEP-susceptible Asn/Asp-containing linkers of varying length were identified (Figure 7). The Asn/Asp-containing linkers were then sorted based on the number of amino acid residues in their linkers. These Asn/Asp-containing linkers formed two major clusters: long and short linkers. The long-linker cluster, containing 13–18 amino acid residues, are found in 32 hololectin linker members that include oats, with OCH1-5 accounting for 15 out of 32 putatively AEP-cleavable and Asn/Asp-containing linkers (Figures 7, 8). In contrast, the short-linker cluster contains 1–6 amino acid residues from 152 hololectin linker members which include wheat, barley and millet (Figures 7, 8). An interesting observation is that short Asn/Asp-containing linkers, but not the long linkers usually contain positive charge amino acids such as Lys and Arg (Figure 7B).

## Discussion

Cereals such as wheat, barley, rice, sorghum, and rye are a rich source of hev-hololectins, but not oats. This study provides an explanation to this divergence based on their biosynthesis. In contrast to many cereal-derived hololectins, oats have cleavable hev-hololectin precursors in which the mature domains of their gene products could be processed by an AEP and then released as small subunits in the form of hev-like peptides, avenatide aV1–aV9. A determining factor is found in their interdomain linker. Oat hev-hololectin precursors, such as OCH1-5, contain long, flexible and putatively AEP-susceptible linkers connecting the tandem-repeating hev-like domains. In contrast, most other cereals containing non-cleavable hololectin precursors have short linkers of 1-6 amino acid residues and which are resistant to bioprocessing. Thus, the interdomain linkers of hololectin precursors provide a key to distinguish cleavable from non-cleavable hololectins.

Avenatide aV1–aV9 with 8 Cys residues are 38-39 amino acids in length. They share a very high sequence similarity to each other (>90%) and a conserved chitin-binding site. Indeed, avenatide aV1 differs only 1 to 3 residues from the other seven members, aV2–aV9. Like other hev-peptides, avenatides are Gly-rich. Together, the 39-residue avenatide aV1 contains a total of 17 Cys and Gly residues, accounting for >43% of its amino acid composition. Like other hev-peptides, avenatides possess an evolutionarily conserved cysteine motif (CX_n_CX_n_CCX_n_CX_n_C) that is arranged in a disulfide-dense cystine-knot framework and a characteristic chitin-binding site as shown in Figure 3 (Rodriguez-Romero et al., 1991; Andersen et al., 1993). Also, like other hev-peptides (Tam et al., 2015; Wong et al., 2016, 2017; Kini et al., 2017; Loo et al., 2021b), avenatide aV1 is antifungal and capable in binding to chitin to inhibit phyto-pathogenic fungal strains. Thus, we can firmly conclude that avenatides belong to the family of hev-peptides based on their size (38–39 amino acids), abundance of glycine and cysteine residues, the presence of the characteristic cysteine motif, and the three essential aromatic amino acid residues which form the chitin-binding site.

The biosynthesis of hev-peptides can be broadly categorized into two major types based on their precursor architectures. The first type (type A) has a three-domain arrangement: a signal peptide, a single hev-peptide domain, and a C-terminal tail which can be short or long (protein-cargo carrying type). Examples of short hev-precursors include morintide mO1 and ginkgotide gB1, both of which contain a short C-terminal tail (<20 amino acids) (Wong et al., 2016; Kini et al., 2017). A subtype of the single-hev-peptide domain precursor is the cargo-carrying hev-peptide precursors with a long C-terminal cargo. Examples are hevein and EeCBP-1 whose precursors are significantly larger, and the C-terminal peptide is a functional protein (Lee et al., 1991; Van den Bergh K. et al., 2002; Van den Bergh K. P. et al., 2002).

**FIGURE 9:**
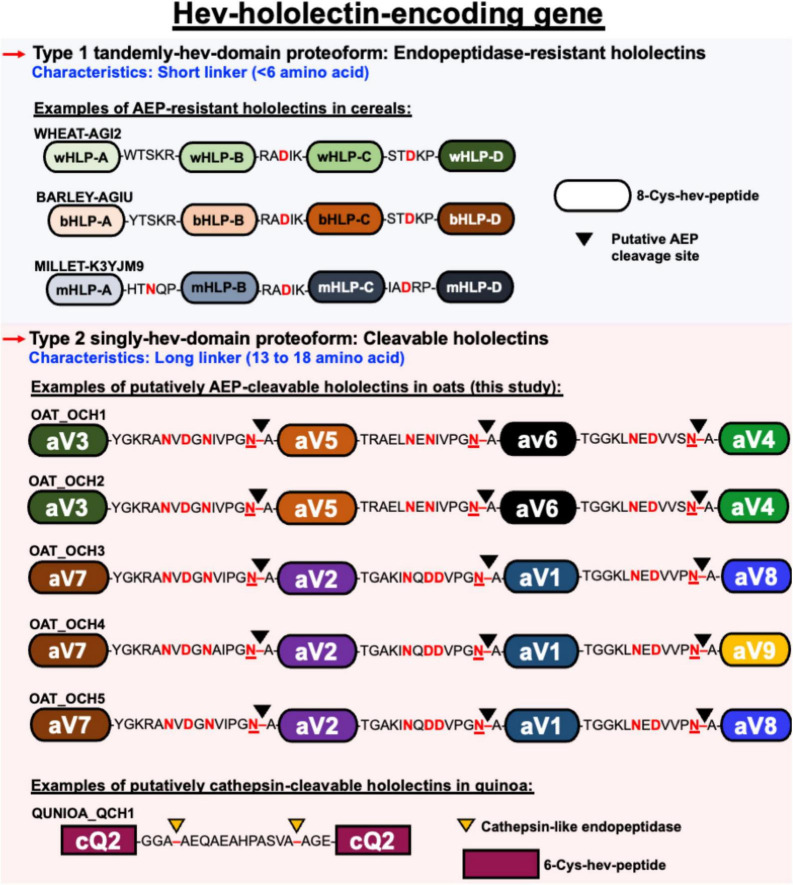
Schematic representation of different architectural types of hev-hololectin. Type 1 proteoforms are endopeptidase-resistant hololectins that are presented as tamdemly hev-domains with short linkers. Endopeptidase-resistant hololectins can be found in wheat, barley and millet. Type 2 proteoforms are cleavable hololectins with long linkers which are presented as singly hev-domain. Putatively AEP-cleavable hololectins with flexible and Asx-rich linkers can be found in oats, and putatively cathepsin-cleavable hololectins with flexible Gly/Ala-rich linker can be found in quinoa.

The second architectural type (type B) belongs to the multi-modular hev-family in which their mature domains contain 2–7 hev-peptide domains (Figure 9). Type B family contains both precursors that are cleavable and non-cleavable (Loo et al., 2021b). Cleavable hev-hololectins are processed by an endopeptidase to release individual hev-peptide domains as hev-peptides. Examples include chenotides from *C. quinoa*, Sm-Amp-1 from *S. media*, and, as demonstrated in this study, avenatides from oats (Slavokhotova et al., 2017; Loo et al., 2021b). The type B precursor architecture, such as avenatide OCH1-5 is hololectin-like (>200 amino acid residues), comprising a signal peptide, four tandem repeats of highly similar hev-peptide domains connected by linkers, and a short C-terminal tail (Wright et al., 1985; Smith and Raikhel, 1989). This precursor architecture and length (>200 amino acid residues) is comparable to the cereal hololectins like WGA, OSA, and phytolacca lectins, all of which contain four hev-peptide domains (Wright et al., 1985; Smith and Raikhel, 1989; Yamaguchi et al., 1995, 1996; Yamaguchi et al., 1997; Zhang et al., 2000). However, the bioprocessing of oat hev-hololectin precursors differs from hololectins such as WGA and OSA, both of which are non-cleavable hev-hololectins. WGA and OSA are expressed as a single-chain multi-modular hev-protein proteoform.

A prominent feature that distinguishes cleavable from non-cleavable hololectins is the length of their linkers, sequences between two cysteinyl residues of two adjacent hev-peptide domains (Figure 9). Non-cleavable hololectins have short linkers that are generally between 1 and 6 amino acids long. In contrast, cleavable hololectins have long linkers of 13–18 amino acids. The avenatide precursors OCH1-5 have linkers of 13–16 amino acids, similar in length to other cleavable hololectins (e.g., chenotides and Sm-Amp-1). The short linkers in non-cleavable hololectins could be crucial to maintain cooperative inter-domain interactions and to improve overall structural protein stability while preventing cleavage by endopeptidases. Long linkers, on the other hand, are likely to be susceptible to proteolytic cleavage, resulting in releasing individual mature tandem-repeating hev-peptide domains as hev-peptides.

The oat precursors OCH1-5 have long linkers that are rich in Asn and Asp (accounting for ∼23% of the total residues) and are susceptible to proteolytic cleavage by AEPs. Our predicted and calculated mass from mass spectrometry for avenatides aV1, aV2, aV4, and aV5 indicate that their N terminus is Ala and cleavage would occur between the conserved Asn-Ala dipeptide at the N-terminus of avenatide precursors. This finding suggests that the hydrolase activity of AEP could be involved in the release of each avenatide. Hololectins such as WGA, barley hololectin and millet hololectin also have Asn/Asp in their linkers which are short, ranging from 3 to 5 amino acids. Such short linkers could hinder AEP access, and in turn, prevent further processing to render them non-cleavable. Overall, avenatide precursors, with tandem-repeating hev-peptide domains, represent the first example of cleavable hololectins that could be bioprocessed by an AEP. Gene amplification through cleavable hololectin precursors could be an evolutionarily advantageous trait to boost the biosynthetic efficiency of these hev-peptide domains and benefit plant survival and reproduction (Panchy et al., 2016).

As functional foods, oats are less likely to cause celiac disease compared to other members of the cereal family (Rashid et al., 2007). Indeed, cereal lectins such as WGA from wheat and glutens are known to trigger celiac disease because the multivalent carbohydrate-binding properties of WGA promote interactions with sialic acid on surface tissues of the gastrointestinal tracts. Such interactions could result in intestinal inflammation and reduce nutrient absorption (De Punder and Pruimboom, 2013). The absence of hev-hololectins by the gut-friendly oats as a functional food could be a distinguish feature among cereals (Wright et al., 1985; Smith and Raikhel, 1989; Mishra et al., 2019). Furthermore, the combination cleavable precursor architecture and the need for AEP as a processing enzyme could increase the biosynthetic efficiency of hev-peptides in oats to provide an evolutionary advantage for plant survival and reproduction.

## Data Availability Statement

The datasets presented in this study can be found in online repositories. The names of the repository/repositories and accession number(s) can be found in the article/Supplementary Material.

## Author Contributions

SL, ST, AK, and WL designed, performed, and analyzed the experiments. SL, AK, and JT wrote the manuscript. All authors reviewed the results and approved the final version of the manuscript.

## Conflict of Interest

The authors declare that the research was conducted in the absence of any commercial or financial relationships that could be construed as a potential conflict of interest.

## Publisher’s Note

All claims expressed in this article are solely those of the authors and do not necessarily represent those of their affiliated organizations, or those of the publisher, the editors and the reviewers. Any product that may be evaluated in this article, or claim that may be made by its manufacturer, is not guaranteed or endorsed by the publisher.

## Footnotes

1
https://www.genome.jp/tools/motif/MOTIF2.html

2
https://www.genome.jp/tools/motif/MOTIF.html
