# Supplementary material for: Hololectin Interdomain Linker Determines Asparaginyl Endopeptidase-Mediated Maturation of Antifungal Hevein-Like Peptides in Oats
Source: Front Plant Sci. 2022 May 10;13:899740. doi: 10.3389/fpls.2022.899740 (PMC9127739; doi:10.3389/fpls.2022.899740)
Supplement: Supplementary file 1 [file Table_1.docx]

Supplementary Table S1. Structural statistics for the final 10 conformers of avenatide aV1^a^

| Distance restraints | |
| --- | --- |
| Intra-residue (*i*-*j* = 0) | 91 |
| Sequential (\|*i*-*j*\| = 1) | 92 |
| Medium range (2 ≤ \|*i*-*j*\| ≤ 4) | 33 |
| Long range (\|*i*-*j*\| ≥ 5) | 44 |
| Hydrogen bond | 8 |
| Total | 268 |
| Average rmsd to the mean structure (Å)^b^ | |
| Backbone atoms  Heavy atoms | 1.23 ± 0.26  1.68 ± 0.27 |
| ϕ/ψ space^c^ | |
| Most favored region (%) | 72.8 |
| Additionally allowed region (%) | 24.8 |
| Generously allowed region (%) | 2.0 |
| Disallowed region (%) | 0.4 |
| RMSD from covalent geometry | |
| Bonds (Å) | 0.0069 ± 0.0023 |
| Angles (deg.) | 0.027 ± 0.027 |
| Impropers (deg.) | 0.374 ± 0.038 |
| RMSD from experimental restraints | |
| NOEs (Å) | 0.0361 ± 0.0030 |
| ^a^ Selected from 100 calculated conformers according to overall energy.  ^b^ Calculated with MOLMOL using range 3-8, 11-37.  ^c^ Calculated with PROCHECK-NMR. | |

Supplementary Table S2. Proton chemical shift assignments for each amino acid residues of avenatide aV1.

|  | **HN (ppm)** | **Hα (ppm)** | **Hβ (ppm)** | | **Others (ppm)** |
| --- | --- | --- | --- | --- | --- |
| A1 |  | 4.252 | 1.563 |  |  |
| C2 | 7.981 | 5.116 | 3.318 | 2.869 |  |
| S3 | 8.429 | 4.303 | 4.178 | 3.966 |  |
| S4 | 8.909 | 4.210 | 4.056 |  |  |
| S5 | 7.973 | 4.585 | 3.932 | 3.766 |  |
| S6 | 7.144 | 4.871 | 3.932 | 3.561 |  |
| P7 |  | 4.541 | 2.201 |  | Hγ, 2.328; Hδ, 3.648, 3.401 |
| C8 | 8.877 | 4.589 | 3.005 | 2.638 |  |
| P9 |  | 4.535 | 1.866 |  | Hγ, 2.267; Hδ, 3.818,3.650 |
| G10 | 8.525 | 3.735 |  |  |  |
| N11 | 8.616 | 4.562 | 2.991 | 2.607 |  |
| Q12 | 6.892 | 4.210 | 1.947 | 1.869 | Hγ, 2.519 |
| C13 | 8.725 | 4.723 | 3.840 | 2.784 |  |
| C14 | 7.925 | 5.198 | 3.036 | 2.744 |  |
| S15 | 9.874 | 4.904 | 4.363 | 4.318 | Hγ, 6.302 |
| K16 | 8.844 | 3.815 | 1.549 | 1.083 | Hγ, 0.533, 0.263 |
| W17 | 7.203 | 4.816 | 3.727 | 2.967 | Hδ1,7.300, Hε1, 10.177 |
| G18 | 8.098 | 4.063, 3.643 |  |  |  |
| Y19 | 7.458 | 4.977 | 3.454 | 2.956 | Hδ, 7.110; H*ε*, 6,841 |
| C20 | 8.824 | 5.710 | 3.085 | 2.838 |  |
| G21 | 8.814 | 3.445, 1.983 |  |  |  |
| L22 | 7.943 | 4.964 | 1.569 |  | Hγ, 1.446; Hδ, 1.036, 0.909 |
| G23 | 7.994 | 4.553, 3.876 |  |  |  |
| G24 | 8.802 | 3.999, 3.814 |  |  |  |
| D25 | 8.953 | 4.485 | 2.483 |  |  |
| Y26 | 7.669 | 4.063 | 2.837 | 2.563 | Hδ, 7.105; H*ε*, 6.741 |
| C27 | 8.269 | 5.139 | 3.576 | 2.766 |  |
| G28 | 7.518 | 4.310, 3.827 |  |  |  |
| S29 | 8.483 | 4.079 | 3.809 | 3.758 |  |
| G30 | 9.095 | 3.954, 3.565 |  |  |  |
| C31 | 7.916 | 4.512 |  |  |  |
| Q32 | 9.646 | 4.480 | 2.093 | 1.467 | Hγ, 2.090, 2.175 |
| S33 | 7.533 | 4.558 | 3.990 |  |  |
| G34 | 8.691 | 4.550, 3.715 |  |  |  |
| P35 |  | 4.791 | 1.779 |  | Hγ, 2.244; Hδ, 3.151 |
| C36 | 8.734 | 4.864 | 3.551 | 2.530 |  |
| T37 | 9.142 | 4.246 |  |  | Hγ2, 1.203 |
| G38 | 8.644 | 3.969, 3.877 |  |  |  |
| A39 | 7.806 | 4.134 | 1.299 |  |  |
