# Supplementary material for: Hololectin Interdomain Linker Determines Asparaginyl Endopeptidase-Mediated Maturation of Antifungal Hevein-Like Peptides in Oats
Source: Front Plant Sci. 2022 May 10;13:899740. doi: 10.3389/fpls.2022.899740 (PMC9127739; doi:10.3389/fpls.2022.899740)
Supplement: Supplementary file 2 [file Data_Sheet_1.docx]

Supplementary Material

**Hololectin interdomain linker determines asparaginyl endopeptidase-mediated maturation of antifungal hevein-like peptides in oats**

Shining Loo ^1,#^, Stephanie V. Tay ^1,#^, Antony Kam ^1^, Warren Lee ^1^, and James P. Tam ^1,*^

^1^ School of Biological Sciences, Nanyang Technological University, Singapore 637551.

^#^ These authors contributed equally to this work

Correspondence: Professor James P. Tam, School of Biological Sciences, Nanyang Technological University, 60 Nanyang Drive, 637551, Singapore. Email: [JPTam@ntu.edu.sg](mailto:JPTam@ntu.edu.sg)


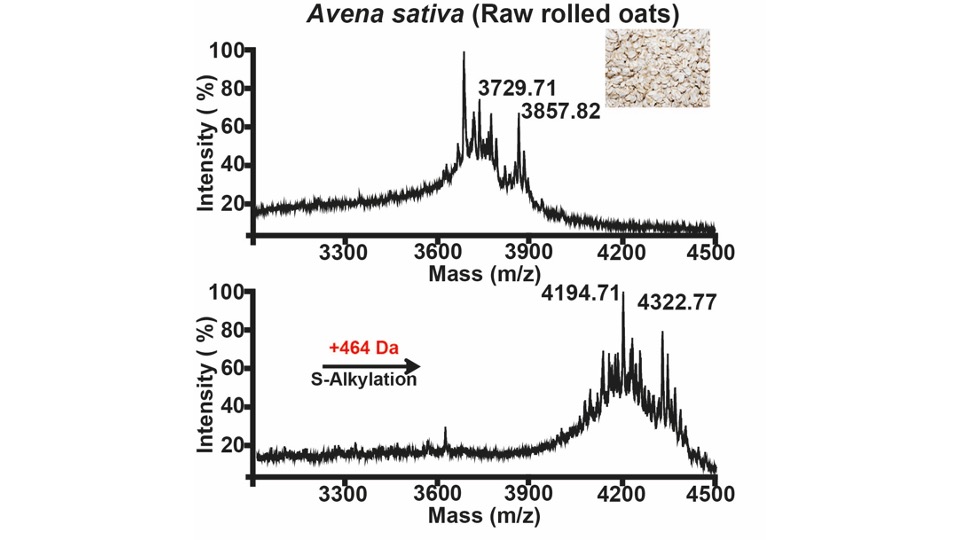


**Supplementary Figure 1.** MALDI-TOF MS profile of aqueous oat extracts and its extract after *S*-reduction by DTT and *S*-alkylation by iodoacetamide (IAM) to give their corresponding linear forms and a gain of 58 Da for each *S*-alkylated Cys. Based on the increase of 464 Da, each cysteine-rich peptide is calculated to contain 8 cysteine residues.


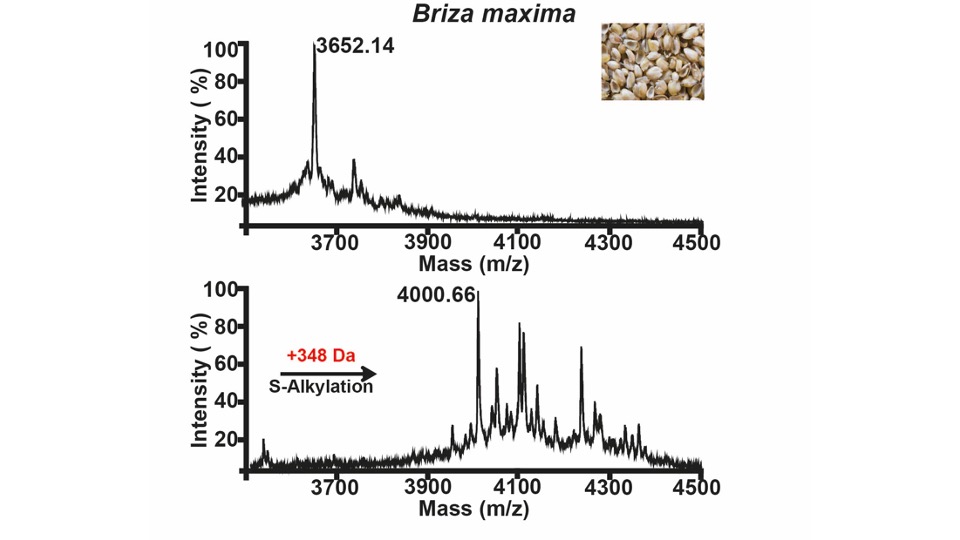
 **Supplementary Figure 2.** MALDI-TOF MS profile of aqueous *Briza maxima* extracts and its extract after *S*-reduction by DTT and *S*-alkylation by iodoacetamide (IAM) to give their corresponding linear forms and a gain of 58 Da for each *S*-alkylated Cys. Based on the increase of 348 Da, each cysteine-rich peptide is calculated to contain 6 cysteine residues.


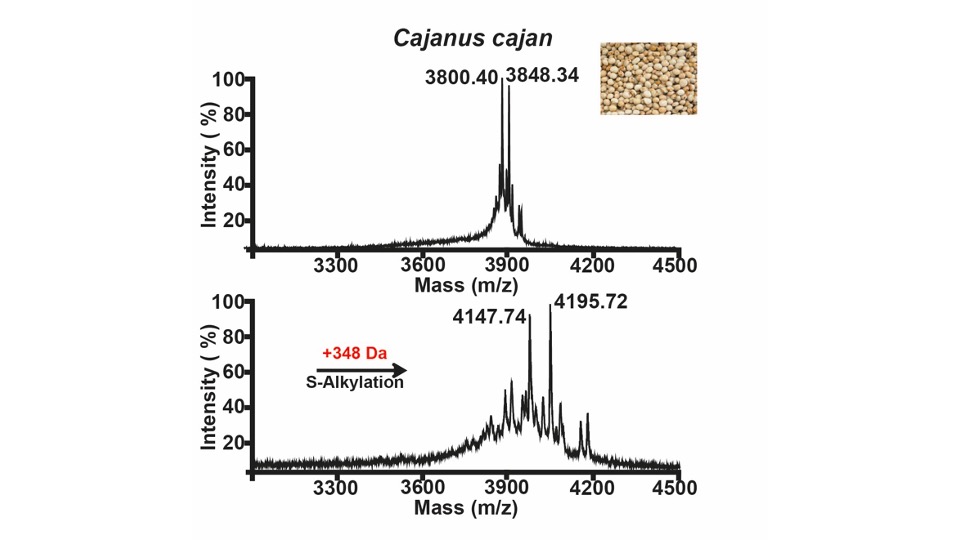
 **Supplementary Figure 3.** MALDI-TOF MS profile of aqueous *Cajanus cajan* extracts and its extract after *S*-reduction by DTT and *S*-alkylation by iodoacetamide (IAM) to give their corresponding linear forms and a gain of 58 Da for each *S*-alkylated Cys. Based on the increase of 348 Da, each cysteine-rich peptide is calculated to contain 6 cysteine residues.


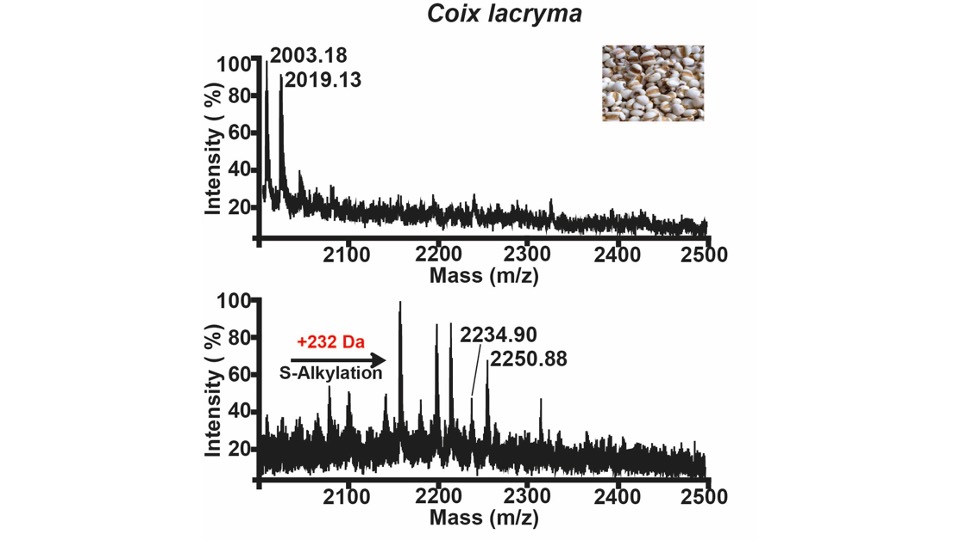
 **Supplementary Figure 4.** MALDI-TOF MS profile of aqueous *Coix lacryma* extracts and its extract after *S*-reduction by DTT and *S*-alkylation by iodoacetamide (IAM) to give their corresponding linear forms and a gain of 58 Da for each *S*-alkylated Cys. Based on the increase of 232 Da, each cysteine-rich peptide is calculated to contain 4 cysteine residues.


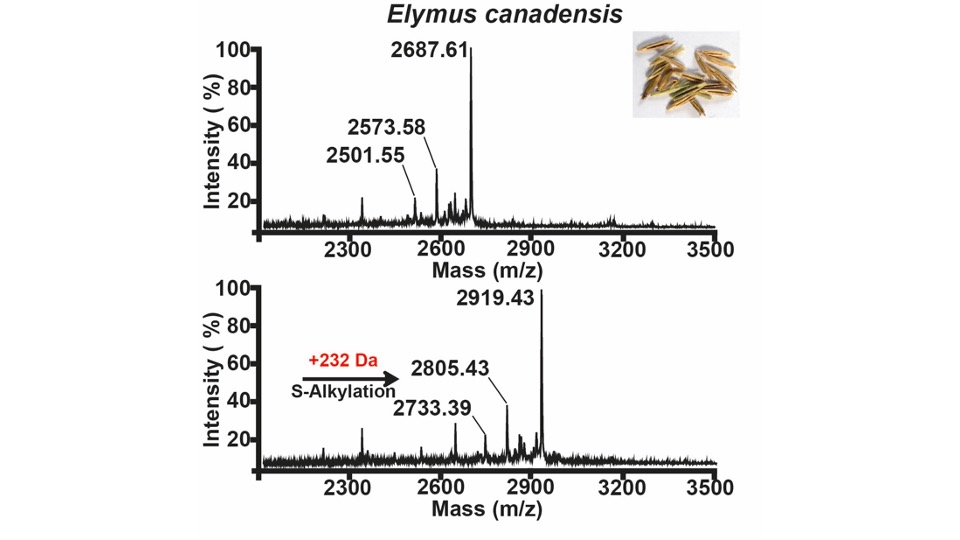
 **Supplementary Figure 5.** MALDI-TOF MS profile of aqueous *Elymus canadensis* extracts and its extract after *S*-reduction by DTT and *S*-alkylation by iodoacetamide (IAM) to give their corresponding linear forms and a gain of 58 Da for each *S*-alkylated Cys. Based on the increase of 232 Da, each cysteine-rich peptide is calculated to contain 4 cysteine residues.


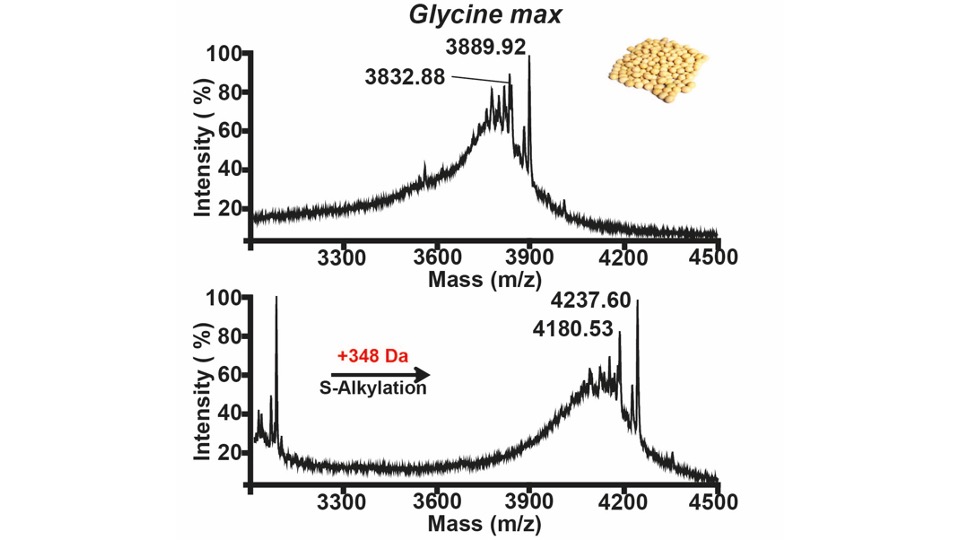
 **Supplementary Figure 6.** MALDI-TOF MS profile of aqueous *Glycine max* extracts and its extract after *S*-reduction by DTT and *S*-alkylation by iodoacetamide (IAM) to give their corresponding linear forms and a gain of 58 Da for each *S*-alkylated Cys. Based on the increase of 348 Da, each cysteine-rich peptide is calculated to contain 6 cysteine residues.


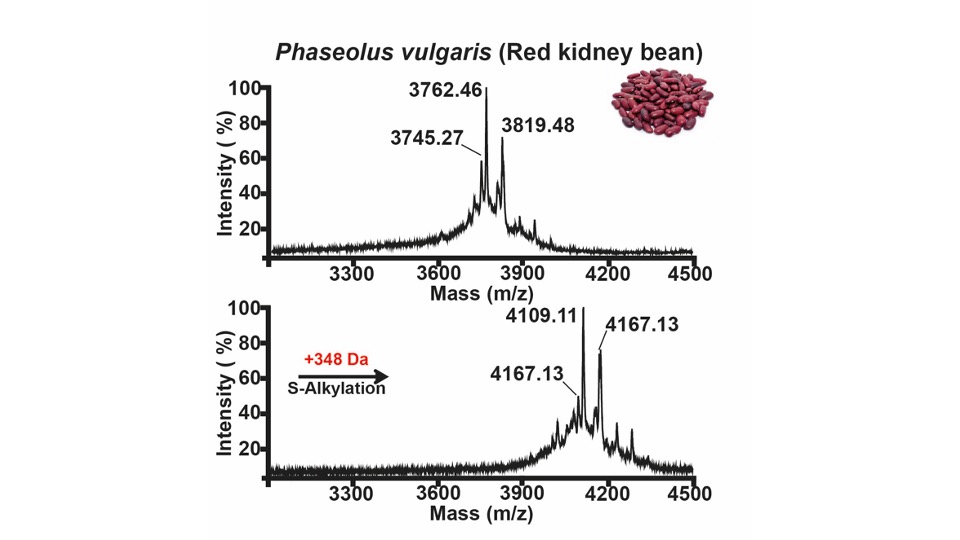
 **Supplementary Figure 7.** MALDI-TOF MS profile of aqueous *Phaseolus vulgaris* extracts (red kidney bean) and its extract after *S*-reduction by DTT and *S*-alkylation by iodoacetamide (IAM) to give their corresponding linear forms and a gain of 58 Da for each *S*-alkylated Cys. Based on the increase of 348 Da, each cysteine-rich peptide is calculated to contain 6 cysteine residues.


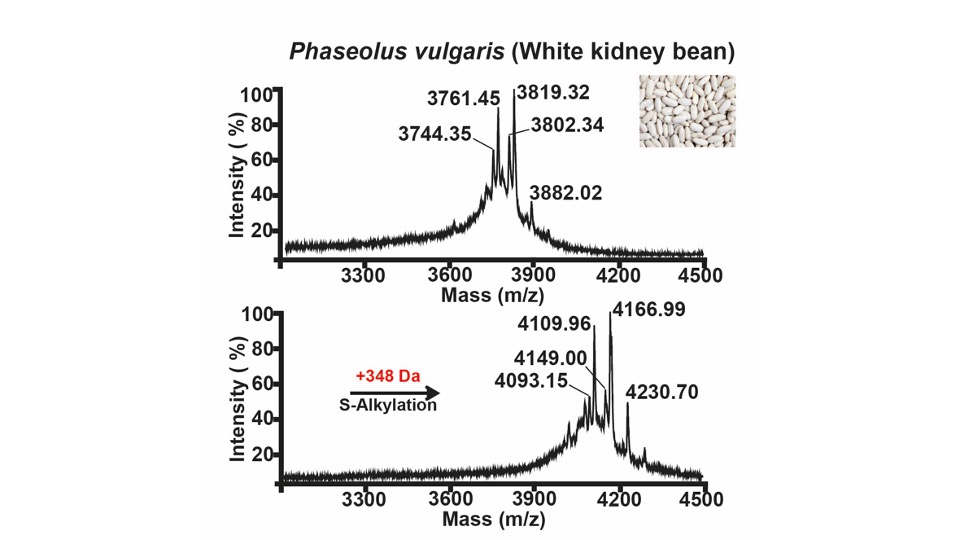
 **Supplementary Figure 9.** MALDI-TOF MS profile of aqueous *Phaseolus vulgaris* extracts (white kidney bean) and its extract after *S*-reduction by DTT and *S*-alkylation by iodoacetamide (IAM) to give their corresponding linear forms and a gain of 58 Da for each *S*-alkylated Cys. Based on the increase of 348 Da, each cysteine-rich peptide is calculated to contain 6 cysteine residues.


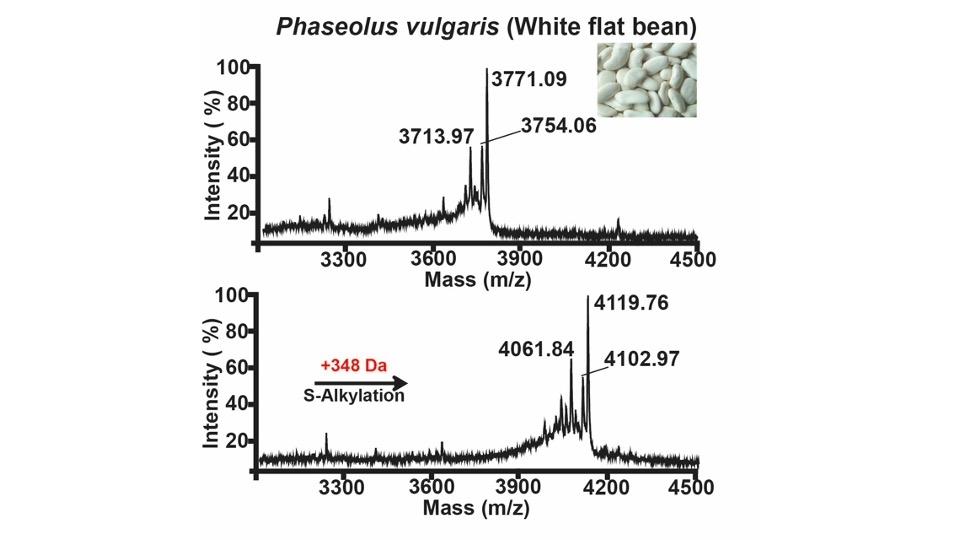
 **Supplementary Figure 10.** MALDI-TOF MS profile of aqueous *Phaseolus vulgaris* extracts (white flat bean) and its extract after *S*-reduction by DTT and *S*-alkylation by iodoacetamide (IAM) to give their corresponding linear forms and a gain of 58 Da for each *S*-alkylated Cys. Based on the increase of 348 Da, each cysteine-rich peptide is calculated to contain 6 cysteine residues.


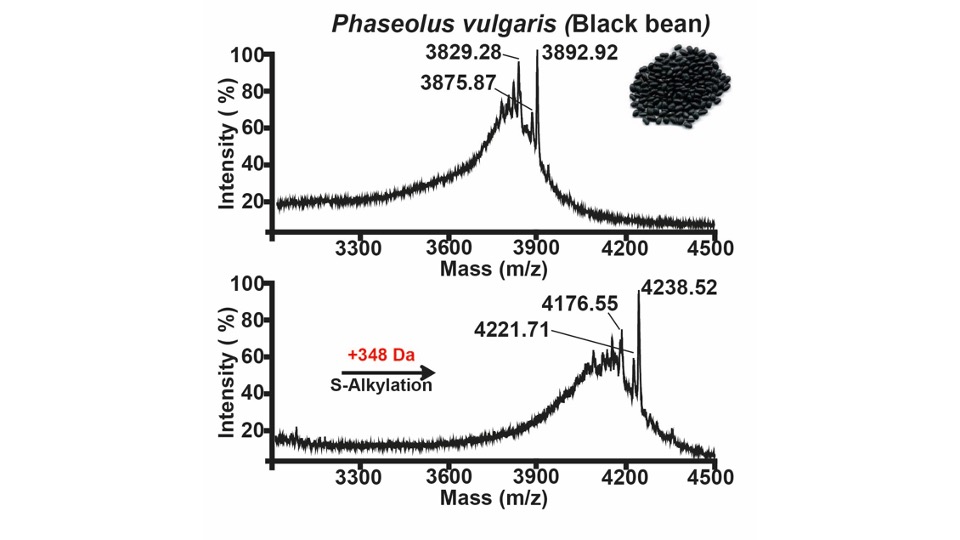
 **Supplementary Figure 11.** MALDI-TOF MS profile of aqueous *Phaseolus vulgaris* extracts (black bean) and its extract after *S*-reduction by DTT and *S*-alkylation by iodoacetamide (IAM) to give their corresponding linear forms and a gain of 58 Da for each *S*-alkylated Cys. Based on the increase of 348 Da, each cysteine-rich peptide is calculated to contain 6 cysteine residues.


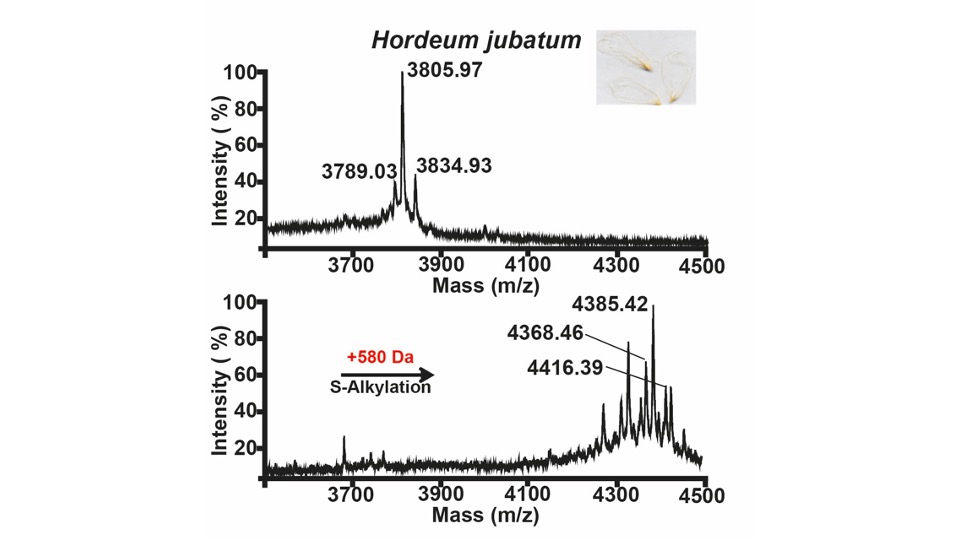
 **Supplementary Figure 12.** MALDI-TOF MS profile of aqueous *Hordeum jubatum* extracts and its extract after *S*-reduction by DTT and *S*-alkylation by iodoacetamide (IAM) to give their corresponding linear forms and a gain of 58 Da for each *S*-alkylated Cys. Based on the increase of 580 Da, each cysteine-rich peptide is calculated to contain 10 cysteine residues.


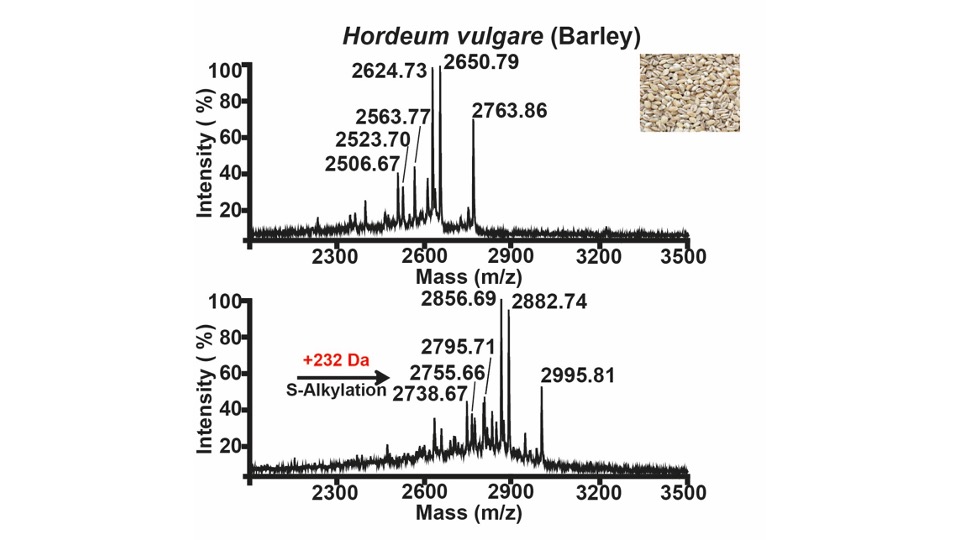
 **Supplementary Figure 13.** MALDI-TOF MS profile of aqueous *Hordeum vulgare* extracts and its extract after *S*-reduction by DTT and *S*-alkylation by iodoacetamide (IAM) to give their corresponding linear forms and a gain of 58 Da for each *S*-alkylated Cys. Based on the increase of 232 Da, each cysteine-rich peptide is calculated to contain 4 cysteine residues.


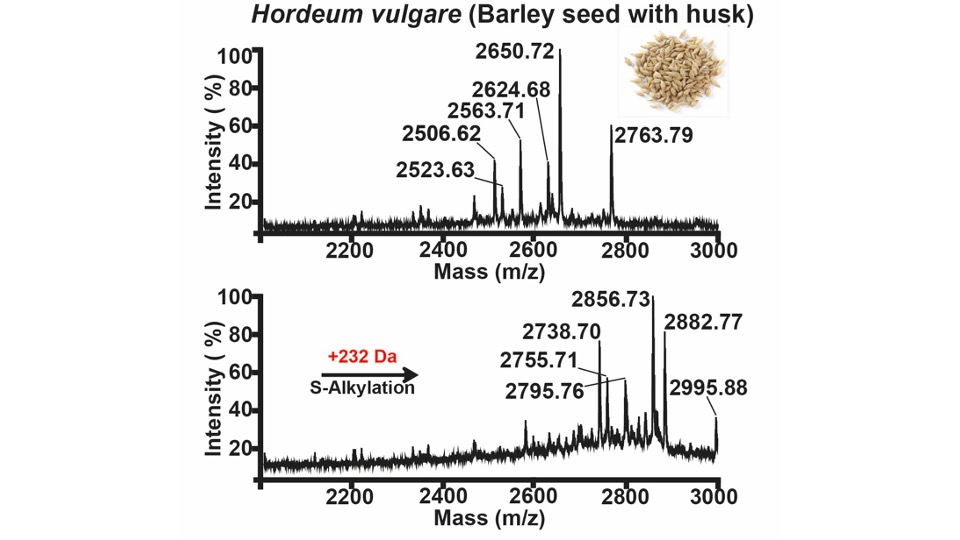
 **Supplementary Figure 14.** MALDI-TOF MS profile of aqueous *Hordeum vulgare* extracts and its extract after *S*-reduction by DTT and *S*-alkylation by iodoacetamide (IAM) to give their corresponding linear forms and a gain of 58 Da for each *S*-alkylated Cys. Based on the increase of 232 Da, each cysteine-rich peptide is calculated to contain 4 cysteine residues.


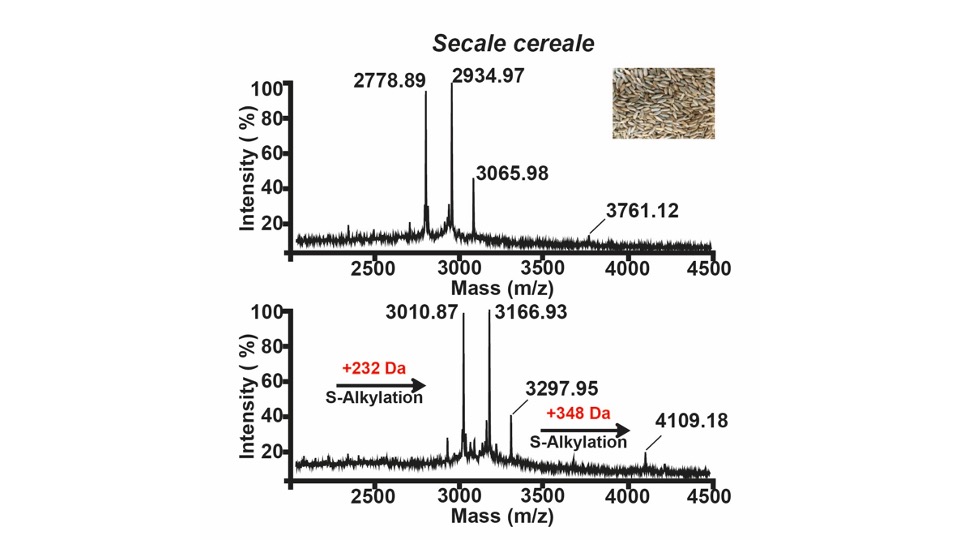
 **Supplementary Figure 15.** MALDI-TOF MS profile of aqueous *Secale cereale* extracts and its extract after *S*-reduction by DTT and *S*-alkylation by iodoacetamide (IAM) to give their corresponding linear forms and a gain of 58 Da for each *S*-alkylated Cys. Based on the increase of 232 and 348 Da, each cysteine-rich peptide is calculated to contain 4 and 6 cysteine residues, respectively.


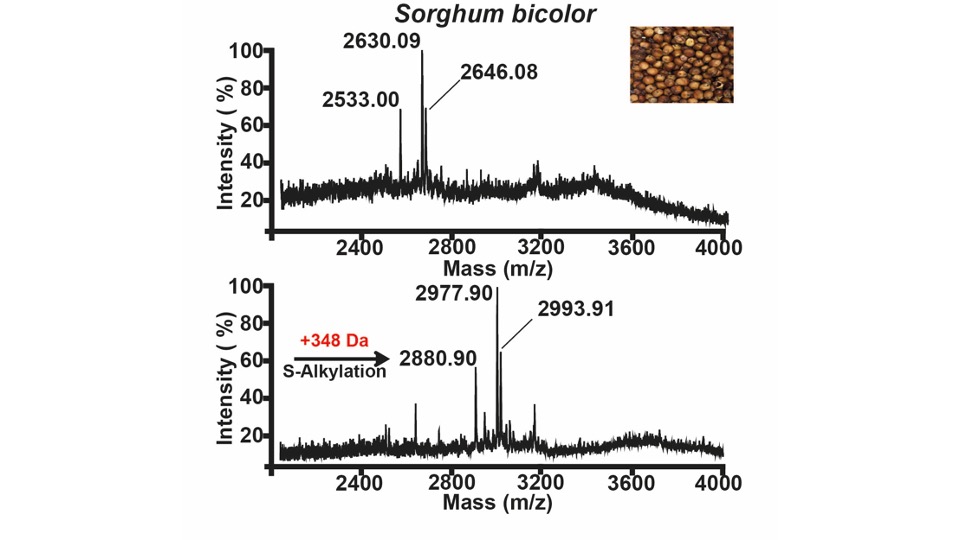
 **Supplementary Figure 16.** MALDI-TOF MS profile of aqueous *Sorghum bicolor* extracts and its extract after *S*-reduction by DTT and *S*-alkylation by iodoacetamide (IAM) to give their corresponding linear forms and a gain of 58 Da for each *S*-alkylated Cys. Based on the increase of 348 Da, each cysteine-rich peptide is calculated to contain 6 cysteine residues.


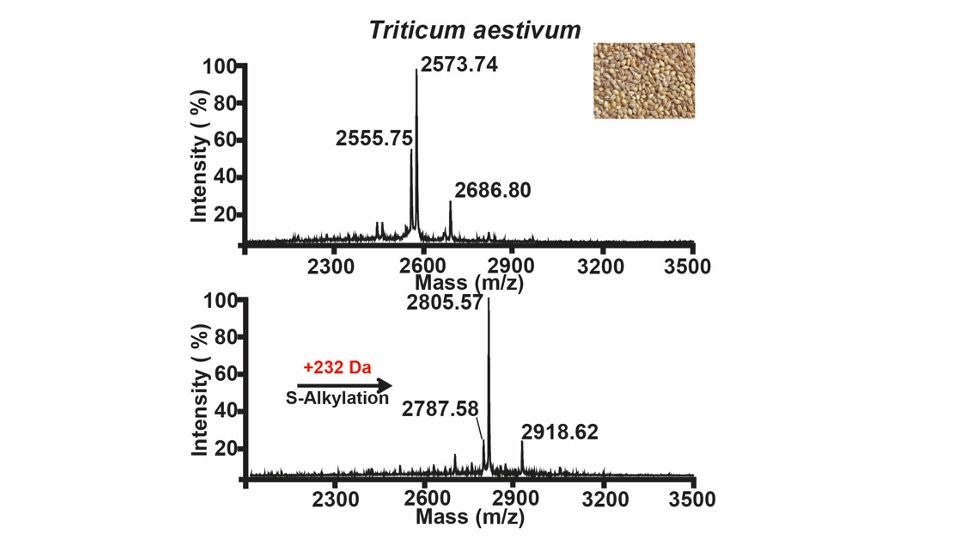
 **Supplementary Figure 16.** MALDI-TOF MS profile of aqueous *Triticum aestivum* extracts and its extract after *S*-reduction by DTT and *S*-alkylation by iodoacetamide (IAM) to give their corresponding linear forms and a gain of 58 Da for each *S*-alkylated Cys. Based on the increase of 232 Da, each cysteine-rich peptide is calculated to contain 4 cysteine residues.


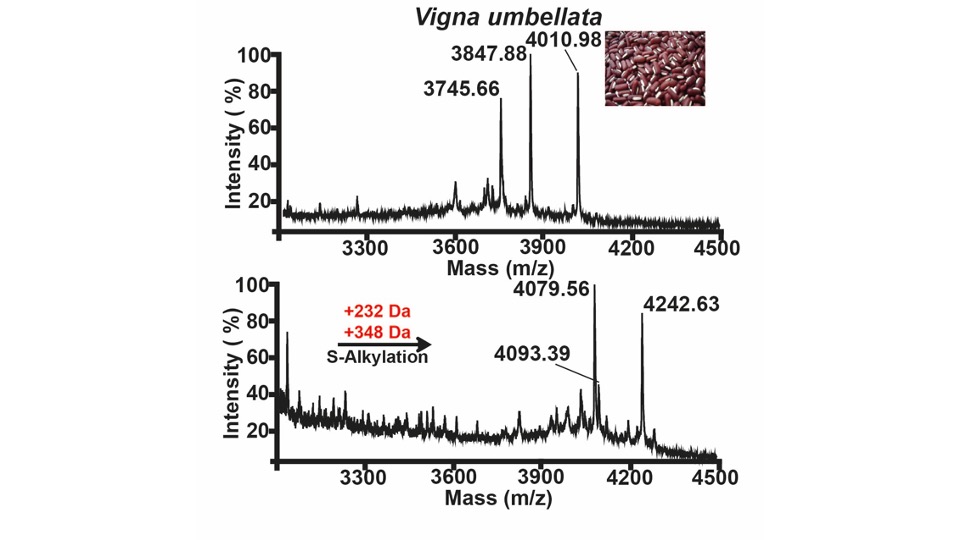
 **Supplementary Figure 16.** MALDI-TOF MS profile of aqueous *Vigna umbellata* extracts and its extract after *S*-reduction by DTT and *S*-alkylation by iodoacetamide (IAM) to give their corresponding linear forms and a gain of 58 Da for each *S*-alkylated Cys. Based on the increase of 232 and 348 Da, each cysteine-rich peptide is calculated to contain 4 and 6 cysteine residues, respectively.


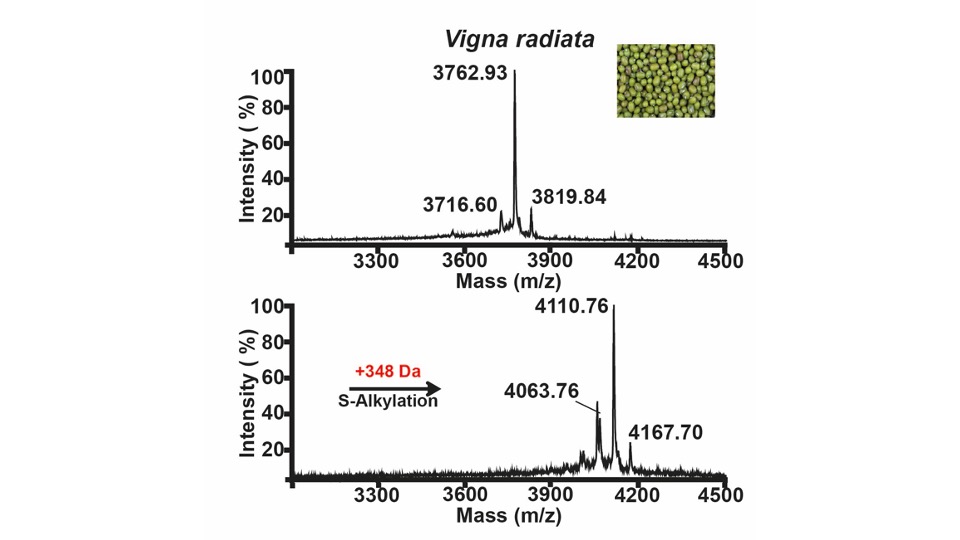
 **Supplementary Figure 17.** MALDI-TOF MS profile of aqueous *Vigna radiata* extracts and its extract after *S*-reduction by DTT and *S*-alkylation by iodoacetamide (IAM) to give their corresponding linear forms and a gain of 58 Da for each *S*-alkylated Cys. Based on the increase of 348 Da, each cysteine-rich peptide is calculated to contain 6 cysteine residues.


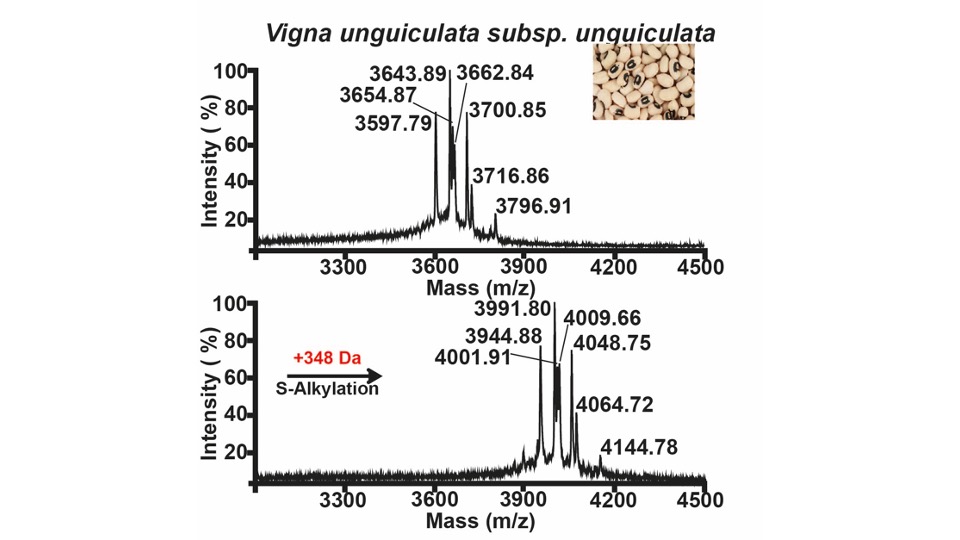
 **Supplementary Figure 18.** MALDI-TOF MS profile of aqueous *Vigna unguiculata* extracts and its extract after *S*-reduction by DTT and *S*-alkylation by iodoacetamide (IAM) to give their corresponding linear forms and a gain of 58 Da for each *S*-alkylated Cys. Based on the increase of 348 Da, each cysteine-rich peptide is calculated to contain 6 cysteine residues.


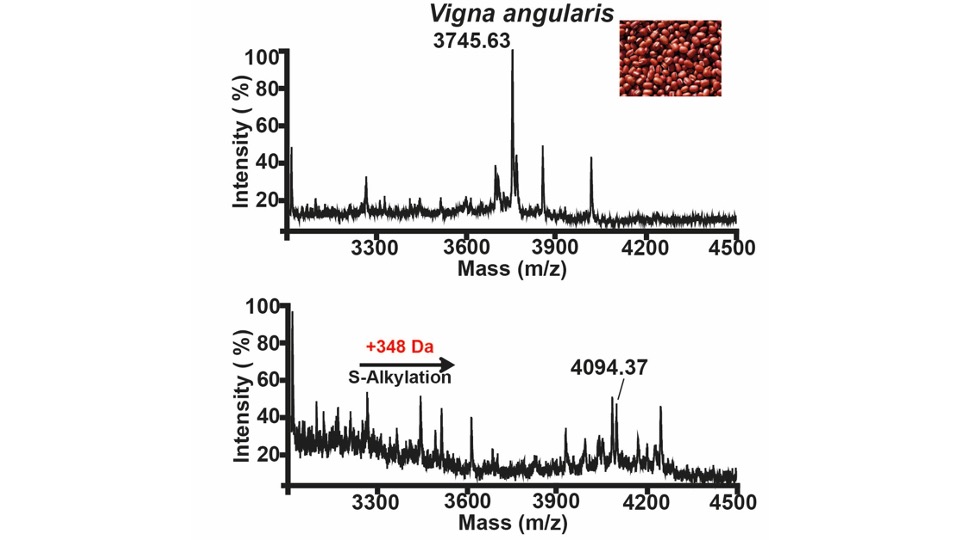
 **Supplementary Figure 19.** MALDI-TOF MS profile of aqueous *Vigna angularis* extracts and its extract after *S*-reduction by DTT and *S*-alkylation by iodoacetamide (IAM) to give their corresponding linear forms and a gain of 58 Da for each *S*-alkylated Cys. Based on the increase of 348 Da, each cysteine-rich peptide is calculated to contain 6 cysteine residues.


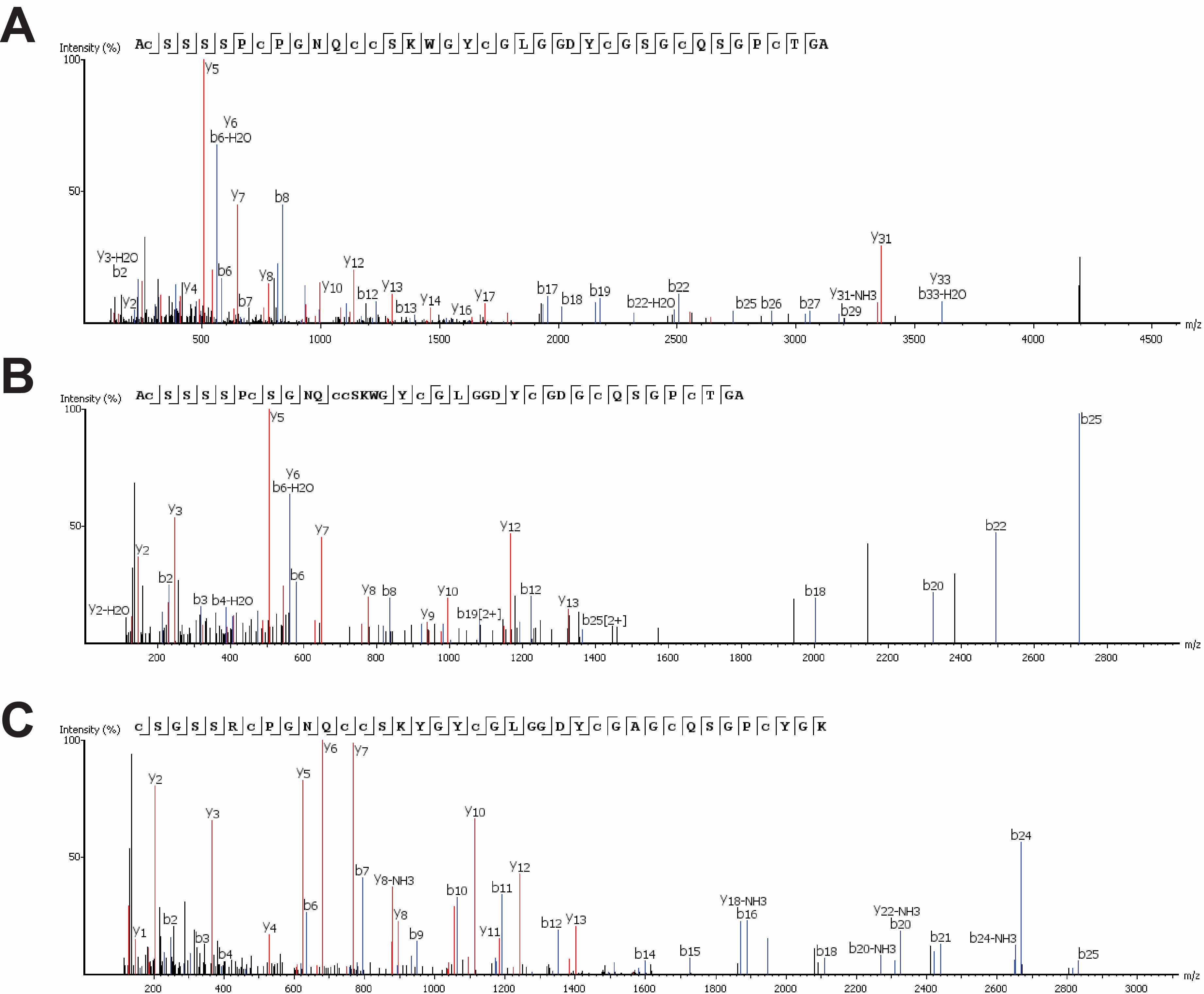


**Supplementary Figure 20.** Peptide sequencing of avenatide aV1-aV3. The purified avenatide aV1-aV3 was *S*-reduced by 20 mM dithiothreitol, and subsequently analyzed by LC-ESI-LTQ-Orbitrap MS/MS and PEAKS studio software. Assignment of isobaric amino acids such as Leu/Ile and Gln/Lys were confirmed by the transcriptome.


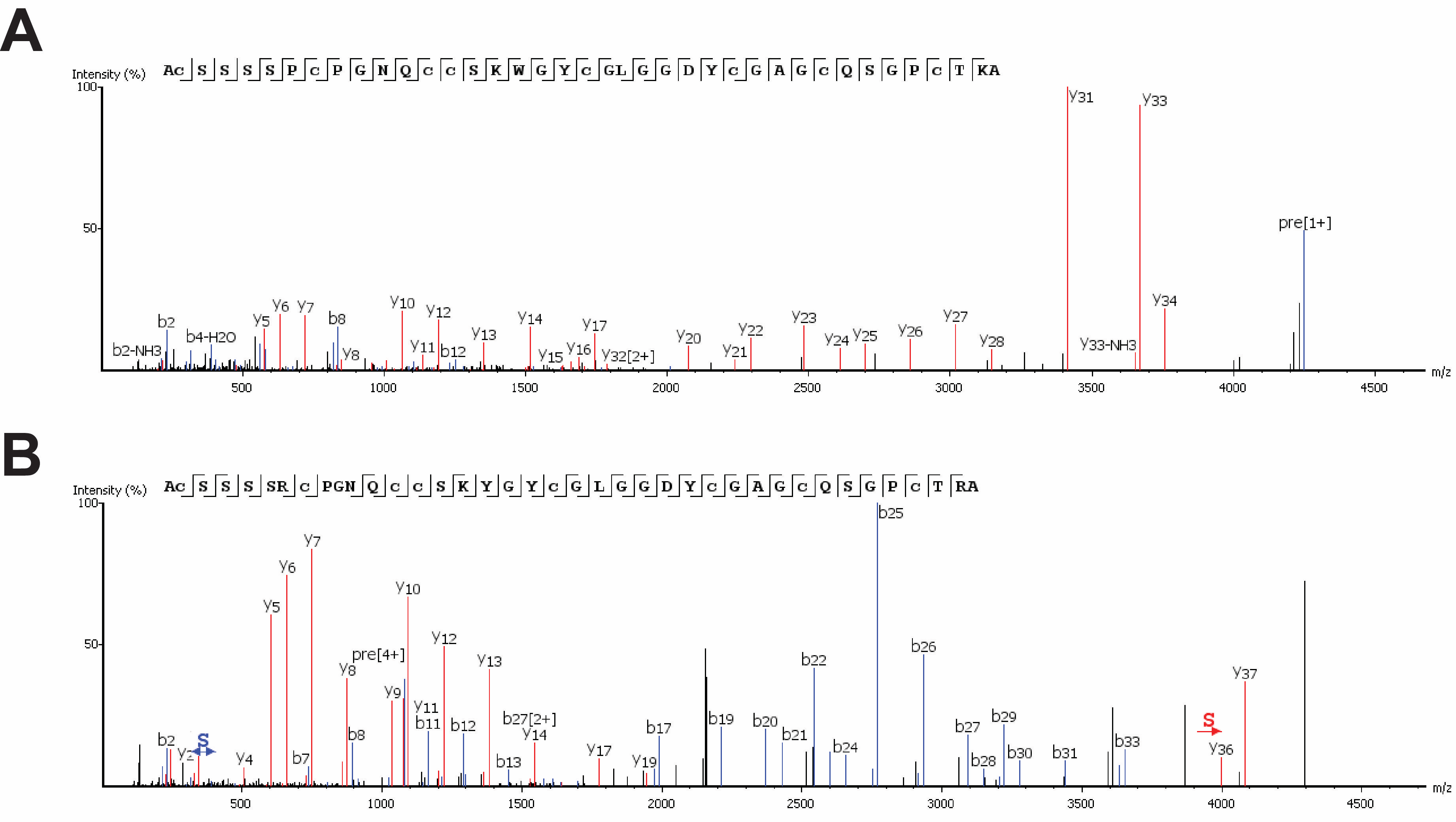


**Supplementary Figure 21.** Peptide sequencing of avenatide aV4-aV5. The purified avenatide aV4-aV5 was *S*-reduced by 20 mM dithiothreitol, and subsequently analyzed by LC-ESI-LTQ-Orbitrap MS/MS and PEAKS studio software. Assignment of isobaric amino acids such as Leu/Ile and Gln/Lys were confirmed by the transcriptome.


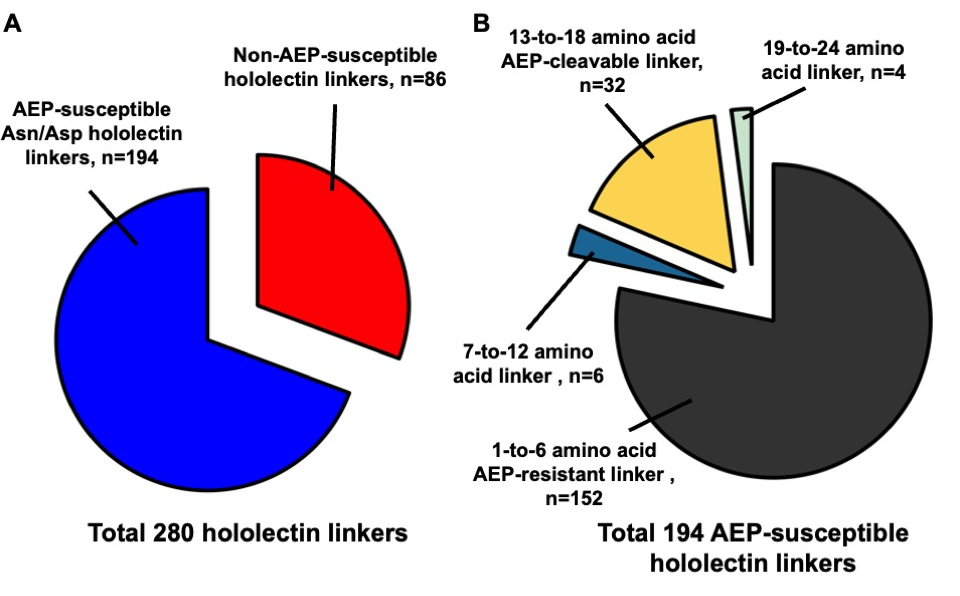


**Supplementary Figure 22.** **(A)** Distribution of 325 linkers of hololectins with Asn/Asp-containing linkers that are susceptible to AEP processing (222 of 325 sequences). **(B)** Distribution of 222 AEP-susceptible linkers that are short, with amino acids <6 amino acids (n=160) and those that are long, with amino acids >13 amino acids (n=54).
