# Supplementary material for: Hololectin Interdomain Linker Determines Asparaginyl Endopeptidase-Mediated Maturation of Antifungal Hevein-Like Peptides in Oats
Source: Front Plant Sci. 2022 May 10;13:899740. doi: 10.3389/fpls.2022.899740 (PMC9127739; doi:10.3389/fpls.2022.899740)
Supplement: Supplementary file 3 [file Data_Sheet_2.docx]

Supplementary Material

**Hololectin interdomain linker determines asparaginyl endopeptidase-mediated maturation of antifungal hevein-like peptides in oats**

Shining Loo ^1,#^, Stephanie V. Tay ^1,#^, Antony Kam ^1^, Warren Lee ^1^, and James P. Tam ^1,*^

^1^ School of Biological Sciences, Nanyang Technological University, Singapore 637551.

^#^ These authors contributed equally to this work

Correspondence: Professor James P. Tam, School of Biological Sciences, Nanyang Technological University, 60 Nanyang Drive, 637551, Singapore. Email: [JPTam@ntu.edu.sg](mailto:JPTam@ntu.edu.sg)

**Supplementary Table S3:** Hololectins with Asn/Asp-containing linkers obtained from data-mining. Accession numbers, species name, category (cereal/non-cereal), precursor sequences and no. of chitin-binding domains (CBD) are indicated:

| **ACCESSION NO.** | **SPECIES** | **CATEGORY** | **PRECURSOR SEQUENCE** | **NO. OF CBD** |
| --- | --- | --- | --- | --- |
| A0A077RWZ6 | Triticum aestivum | cereal | MMSNKALTLGAVVVLAFAMAGAHAEQCGHAADGMECPNNLCCSAWGYCGMDANYCGDGCQSGACYEPKRCGAQAGANAVTCPNNHCCSGDGFCGYGQEYCGAGCQNGPCRANIKCSADKPCLSNFCCSQYGYCGLGVEFCGQGCQSGACHDAVGAAALPLSSIVQG | 3 |
| A0A2G2V3P5 | Capsicum baccatum | non-cereal | MRGSSALVSLLALALFLLLNVSANPSHPFHLPANEMILGQLELAVAPGACGEQAGGKECPSGLCCAQWGFCGSGPEYCGVLETRQSQWEEPAETDLCGIQSGGKRCPSGQCCTVFGLCTAAPDMCKHFCQTSLAGVRLATFVIRQCNMPALPIPDRRCGIQAGGLYCPIGQRTLKSL | 2 |
| A0A4S8J4P4 | Musa balbisiana | non-cereal | MASLLFCLECRKMKLPFVVIFGLGSLLGSLAQQCGPAAGGKTCLDGLCCSKYGYCGSTFAYCNDSGYQCGSQAAGGVCSAWQCCSQHGYCGNTSDYCGSGCQSQCDGGSGSGDSDAQCGSQAAGALCSDGQCCSQYGYCGTTSDYCGVSRKSTTWTHKEFASISISVMLCG | 3 |
| ABE77384.1 | Triticum aestivum | cereal | MKGLLLCALALAFAAVTTHAQLQSCPTRCGKQADGMECPNNLCCSKDGYCGLGVDYCSAGAGCQSGACYDNKICGAQANGTLCRNNHCCSSGGRCGYGREYCSNGCQGGPCWADLKCGHLDNGKLCPNNLCCSQYGYCGLGPEFCGTGCQNGACSTDKPCGNKANGAPCTNNYCCSQYGSCGLGKDYCGTGCQNGACN | 4 |
| ABIJ-2008792 | Selaginella lepidophylla | non-cereal | MALIFALLMLISAGTRAQDCGSQASFASCPPAQCCSQYGYCGTTAAYCGSGCQSQCNQQMCGIQGNFAACPSSSCCSQYGFCGTGSAYCGQGCQSGACPSTPVTPVKIGYWYVDSSPASNINSCLYTHLLYAFVDLNPTTFSVAPSPNLDPGNSKISSFVSTVKAKNPSIKTLISIGGGSSSKDAFAAMVSSSSSRAAFINSTITLANRYGFDGLD | 2 |
| P10968_AGI1_WHEAT | Triticum aestivum | cereal | MKMMSTRALALGAAAVLAFAAATAQAQRCGEQGSNMECPNNLCCSQYGYCGMGGDYCGKGCQNGACWTSKRCGSQAGGATCTNNQCCSQYGYCGFGAEYCGAGCQGGPCRADIKCGSQAGGKLCPNNLCCSQWGFCGLGSEFCGGGCQSGACSTDKPCGKDAGGRVCTNNYCCSKWGSCGIGPGYCGAGCQSGGCDGVFAEAITANSTLLQE | 4 |
| P02876_AGI2_WHEAT | Triticum aestivum | cereal | MRKMMSTMALTLGAAVFLAFAAATAQAQRCGEQGSNMECPNNLCCSQYGYCGMGGDYCGKGCQNGACWTSKRCGSQAGGATCPNNHCCSQYGHCGFGAEYCGAGCQGGPCRADIKCGSQSGGKLCPNNLCCSQWGFCGLGSEFCGGGCQSGACSTDKPCGKDAGGRVCTNNYCCSKWGSCGIGPGYCGAGCQSGGCDAVFAGAITANSTLLAE | 4 |
| AL820037.1 | Triticum aestivum | cereal | ARGSTMALTLGAAVFLAFAAATAQAQRCGEQGSNMECPNNLCCSQYGYCGMGGDYCGKGCQNGACWTSKRCGSQAGGATCPNNHCCSQYGHCGFGAEYCGAGCQGGPCRADIKCGSQSGGKLCPNNLCCSQWGFCGLGSEFCGGGCQSGACSTDKPCGKDAGGRVCTNNYCCSKWGSCGIGPGYCGAGCQSGGCDAVF | 4 |

**Supplementary Table S3 (cont’):** Hololectins with Asn/Asp-containing linkers obtained from data-mining. Accession numbers, species name, category (cereal/non-cereal), precursor sequences and no. of chitin-binding domains (CBD) are indicated:

| **ACCESSION NO.** | **SPECIES** | **CATEGORY** | **PRECURSOR SEQUENCE** | **NO. OF CBD** |
| --- | --- | --- | --- | --- |
| BAA02709.1_synthetic_construct | Triticum aestivum | cereal | MRLLLLLLLLPLALGQRCGEQGSNMECPNNLCCSQYGYCGMGGDYCGKGCQNGACWTSKRCGSQAGGATCPNNHCCSQYGHCGFGAEYCGAGCQGGPCRADIKCGSQSGGKLCPNNLCCSQWGFCGLGSEFCGGGCQSGACSTDKPCGKDAGGRVCTNNYCCSKWGSCGIGPGYCGAGCQSGGCDAVFAGAITANSTLLAE | 4 |
| BG365763.1 | Hordeum vulgare subsp. Vulgare | cereal | KTRRMKMMSTRALALGAAAVLAFAAATAHAQRCGEQGSNMECPNNLCCSQYGYCGMGGDYCGKGCQNGACYTSKRCGTQAGGKTCPNNHCCSQWGYCGFGAEYCGAGCQGGPCRADIKCGSQAGGKLCPNNLCCSQWGYCGLGSEFCGEGCQGGACSTDKPCGKAAGGKVCTNNYCCSKWGSCGIGPGYCGAGCQSGGCDGVFADAIAANSTLVAE | 4 |
| BQ246423.1 | Triticum aestivum | cereal | KCTTSTKKTKSMKMMSTRALALGAAAVLAFAAATAQAQRCGEQGSNMECPNNLCCSQYGYCGMGGDYCGKGCQNGACWTSKRCGSQAGGATCTNNQCCSQYGYCGFGAEYCGAGCQGGPCRADIKCGSQAGGKLCPNNLCCSQWGFCGLGSEFCGGGCQSGACITDKPC | 4 |
| CD901987.1 | Triticum aestivum | cereal | PRVRAQPAPREIMRKMMSTMALTLGAAVFLAFAAATAQAQRCGEQGSNMECPNNLCCSQYGYCGMGGDYCGKGCQNGACWTSKRCGSQAGGATCPNNHCCSQYGHCGFGAEYCGAGCQGGPCRADIKCGSQSGGKLCPNNLCCSQWGFCGLGSEFCGGGCQSGACSTDKPCGKDAGGRVCTNNYCCSKWGSCGIGPGYCGAG | 4 |
| CHJJ-2124255 | Lejeuneaceae sp | non-cereal | MIAASISLVLLAFASSVAGNCLVGGRCGLGQCCSAYGFCGSGAQYCGAVTVVQPAPVYATRDCRVVGCSAGYCCSSFGYCGTTSEYCGIGAPAPAVGSCRLTGCPAGSCCSSYG | 2 |
| CJ776108.1 | Triticum aestivum | cereal | STREEVKRKKMMSTRALALGVSVVLAFAVTTHAQRCGEQGSGMECPNNLCCSQYGYCGMGGDYCGNGCQNGACYTSKRCGSQAGGAVCPNNHCCSQYGHCGFGSEYCGAGCQGGPCRADIKCGSQDGGKLCPNNLCCSQWGYCGLGSEFCGVGCQNGACSTDKPCGKNAGGRACTNNYCCSQWGSCGIGPAYCGAGCQSGGCGAVFAEAIAANSTSTLLKE | 4 |
| CJ777748.1 | Triticum aestivum | cereal | KKMMSTRALALGAAVVLAFAAATAHAQRCGEQGSGMECPNNLCCSQYGYCGMGGDYCGNGCQNGACYTSKRCGTQAAGATCPNNHCCSQYGHCGFGEEYCGAGCQGGPCRANIKCGSQAGGKLCPNNLCCSQWGYCGLGSEFCSNGCQSGACSSSKPCGKDNGGRVCTNNYCCSQWGHCGIGPGYCGAGCQSGGCXAVFADAITANSTLLRE | 4 |
| CK169134.1 | Triticum aestivum | cereal | RGRGRGHILAMKGLLLCALALAFAAVTTHAQLQSCPTRCGKQADGMECPNNLCCSKDGYCGLGVDYCSAGAGCQSGASYDNKICGAQANGTLCRNNHCCSSGGRCGYGREYCSNGCQGGPCWADLKCGHLDNGKLCPNNLCCSQYGYCGLGPEFCGTGCQNGACSTDKPCGNKANGAPCTNNYCCSQYGSCGLGKDYCGTGCQNGACN | 4 |

**Supplementary Table S3 (cont’):** Hololectins with Asn/Asp-containing linkers obtained from data-mining. Accession numbers, species name, category (cereal/non-cereal), precursor sequences and no. of chitin-binding domains (CBD) are indicated:

| **ACCESSION NO.** | **SPECIES** | **CATEGORY** | **PRECURSOR SEQUENCE** | **NO. OF CBD** |
| --- | --- | --- | --- | --- |
| EG378972.1 | Leymus cinereus | non-cereal | YTQPKKKEEKVKMLNKAPLTLFAAVVLAFAAVAAHGQLCGVDGDGMECPRNLCCSQWGYCGLGVNYCGNGCQSGACYTSKRCGKQAGNATCPNNYCCSKYGYCGLGAEYCGDGCQGGPPCRANIKCDIDKLCNNNLCCSQWGYCGLGSEYCGTGCQSGACSTDKPCGSGTPCTNNYCCSRNGSCGVGSDYCGAGCQSGGCYAFLADAIAVTAETNFTLQAE | 4 |
| EG395772.1 | Leymus cinereus | non-cereal | YTQPKKKEEKVKMLIKAPLTLFAAVVLAFAAVAAHGQLCGVDGDGMECPRNLCCSQWGYCGLGVNYCGNGCQSGACYTSKRCGKQAGNATCPNNYCCSKYGYCGLGAEYCGDGCQGGPPCRANIKCDIDKLCNNNLCCSQWGYCGLGSEYCGTGCQSGACSTDKPCGSGTPCTNNYCCSRNGSCGVGSDYCGAGCQSGGCYAFLADAIAVTAETNFTLQAE | 4 |
| EG396761.1 | Leymus cinereus | non-cereal | EKVKMLIKAPLTLFAAVVLAFAAVAAHGQLCGVDGDGMECPRNLCCSQWGYCGLGVNYCGNGCQSGACYTSKRCGKQAGNATCPNNYCCSKYGYCGLGAEYCGDGCQGGPPCRANIKCDIDKLCNNNLCCSQWGYCGLGSEYCGTGCQSGACSTDKPCGSGTPCTNNYCCSRNGSCGVGSDYCGAGCQXGGCYAFLADAIAVTAETNFTLQAE | 4 |
| EMS45413.1 | Triticum urartu | non-cereal | MKGLLLCALALAFAVVTTHAQLQYCEKRCGKQADGMECPNNLCCSKDGYCGLGFDYCSAAAGCQSGACYNNKICGTQAGGAFKYGYCGLGPEFCGAGCQNGACSTDKPCGNQANGARCTNNYCCSQYGSCGLGKDYCGTNCQSGACYNDSILANILKCVP | 2 |
| EMS53669.1 | Triticum urartu | non-cereal | MMSTKALTLGAVVVLAIAMAGAHAEQCGHAADGMECPNNLCCSAWGYCGMDANYCGDGCQSGACYEPKRCGAQAEGNAVTCPNNHCCSGNGYCGYGQEYCGDGCQNGPCRANIKCSADKPCLSNFCCSKYGYCGLGVEFCGQGCQSGACHDAVGAAALPLSSIVQG | 3 |
| EMS64751.1 | Triticum urartu | non-cereal | MMSTRALALGVAVVLAFAVTTHAQRCGEQGSGMECPNNLCCSQYGYCGMGGDYCGNGCQNGACYTSKRCGSQAGGKLCPNNLCCSQWGYCGLGSEFCGVGCQNGACSTDKPCGKNAGGRACTNNYCCSQWGSCGIGPAYCGAGCQSGGCGAVFAEAIAANSTSTLLKE | 3 |
| EMS64753.1 | Triticum urartu | non-cereal | MKMMSTRALVLGAAAVLAFAAATAQAQRCGEQGSNMECPNNLCCRPQTGGKLCPNNLCCSQWGFCGLGSEFCGGGCQSGACSTDKPCGKDAGGRVCTNNYCCSKWGSCGIGPGYCGAGCQSGGCDGVFAEAITANSTLLQE | 2 |
| FF351450.1 | Pseudoroegneria spicata | non-cereal | PAPRGKMMKMMSTRALALGAAAVLAFAAATAHGQRCGEQGSGMECPNNLCCSQYGYCGMGGDYCGKGCQNGACYTSKRCGTQAGGKTCPNNHCCSQYGHCGFGSEYCGAGCQGGPCRADIKCGSQAGGKLCPNNLCCSQWGYCGLGSEFCGEGCQNGACSTDKPCGKDGGGRVCTNNYCCSKWGSCGIGPAYCGAGCQSGGCDAVFAEAIDANSTLLAE | 4 |

**Supplementary Table S3 (cont’):** Hololectins with Asn/Asp-containing linkers obtained from data-mining. Accession numbers, species name, category (cereal/non-cereal), precursor sequences and no. of chitin-binding domains (CBD) are indicated:

| **ACCESSION NO.** | **SPECIES** | **CATEGORY** | **PRECURSOR SEQUENCE** | **NO. OF CBD** |
| --- | --- | --- | --- | --- |
| FF356830.1 | Pseudoroegneria spicata | non-cereal | QPAPRGKMMKMMSTRALALGAAAILAFAAATAHGQRCGEQGSGMECPNNLCCSQYGYCGMGGDYCGKGCQNGACYTSKRCGTQAGGKTCPNNHCCSQYGHCGFGAEYCGAGCQGGPCRADIKCGSQAGGKLCPNNLCCSQWGYCGLGSEFCGEGCQNGACSTDKPCGKDGGGRVCTNNYCCSKWGSCGIGPAYCGAGCQSGGCDAVFAEAIDANSTLLAE | 4 |
| FF367588.1 | Pseudoroegneria spicata | non-cereal | MMSTRALALGAAAVLAFAAATAHGQMCGEQGSGMECPNNLCCSQYGYCGMGGDYCGKGCQNGACYTSKRCGTQAGGKTCPNNHCCSQYGHCGFGAEYCGAGCQGGPCRADIKCGSQAGGKLCPNNLCCSQWGYCGLGSEFCGEGCQNGACSTDKPCGKDGGGRVCTNNYCCSKWGSCGIGPAYCGTGCQSGGCDAVFAEAIDANSTLLA | 4 |
| gnl\|onekp\|CMEQ_scaffold_2080348 | Orthotrichum lyellii | non-cereal | ILLIIVAVKAGVVEGAEVGSCGVLNGGKICDQGNCCSEANFCGISSAHCGAGCQLEYTPIGGCEGDTRTGPTSAPGATTPMCGKSVGNAKCKDNLCCSKNGFCGTTGSYCYSTEGCQSQC | 2 |
| gnl\|onekp\|IGUH_scaffold_2164028 | Schwetschkeopsis fabronia | non-cereal | ILIASVFAAAAAAQNCGPSYGNQKCAAGKCCSQYGWCDTGAAYCDPATCLKTYSGTGSKCAAPSDPYASTVPNIDVCGKAQGGVSCPGAGKDKYFYRCCSSQGHCGPKNNIQDQAIYCGAGCQAGFGDCSTNRKKPADPTTPPKTANAGDTCGPIVNAKCATGLCCSGSNFCGTGADFCGAANWCQSKWGKCS | 3 |
| gnl\|onekp\|JADL_scaffold_2042368 | Rhynchostegium serrulatum | non-cereal | MASRILLLAALTLAMASTALGAECGHQAGDAVCLFRACCSKDGMCGRGEDYCGDGCQEDAGHCDKGTVAASAECGHQAGDAVCLSGACCSKDGMCGRGEDYCGIGCQEDAGRCNKGRTATTGAECGSQAGDAVCLFGACCSKYGMCGRGEDYCGDGCQEDA | 3 |
| gnl\|onekp\|LNSF_scaffold_2010351 | Hypnum subimponens | non-cereal | MASRILLLAALTLAMAFTAFGAECGHQAGDAVCLFGACCSKDGMCGRDENYCGDGCQKDAGRCNVAEGTTVDAASAAECGHQAGDAVCLFGACCSKDGMCGREEEYCGDGCQEDAGRCNKGSTYTATTDAECGSQAGDAMCLFGACCSENGMCGHGEDYCGDGCQKDA | 3 |
| gnl\|onekp\|QWFV_scaffold_2016436 | Bambusina borreri | non-cereal | AVLLLMLAACSSAQAQNCGPTLKVSCASGLCCSQYNYCGADPAHCSSGCQPLYGVCSAPGPQAPPPLISSGTCGASNNMSCPSGLCCSQYNYCGVDPAHCGSGCQPQYGHCSAPSPESGQSPPTPSSDGECGASNKLSCSSGLCCSQYNFCGSDSAHCGSGCQPEWGQCFPLK | 3 |
| gnl\|onekp\|TAVP_scaffold_2078043 | Calliergon cordifolium | non-cereal | LLLAALTLVMAFTALGAECGHQAGDAVCLFGACCSKDGLCGREEEYCGDGCQEDAGRCNKGSTAPMDAECGSQAGDAVCLFGACCSKDGMCGHGEDYCGNGCQKDAGRC | 2 |
| gnl\|onekp\|YNFJ_scaffold_2022557 | Microtea debilis | non-cereal | METKASFTILALLLILSPTIITGQRCGSQFGGSVCPNGNCCSKWGWCGNTGEYCFPQNCQSRCGDSRCGGQAWGRVCPYNQCCSSSGWCGTTPAYCTGGCQSQCRRALAAG | 2 |

**Supplementary Table S3 (cont’):** Hololectins with Asn/Asp-containing linkers obtained from data-mining. Accession numbers, species name, category (cereal/non-cereal), precursor sequences and no. of chitin-binding domains (CBD) are indicated:

| **ACCESSION NO.** | **SPECIES** | **CATEGORY** | **PRECURSOR SEQUENCE** | **NO. OF CBD** |
| --- | --- | --- | --- | --- |
| gnl\|onekp\|YNFJ_scaffold_2173610 | Microtea debilis | non-cereal | RMETKASVAILALFLILSPTFIAGQQCGSQAGGRTCPNGNCCSQWGWCGNTDDYCAGGCQSQCGSNRCGRDFWGRVCSSNRCCSAWGWCGTDSLACGSGCQSQCHGLGLPTPV | 2 |
| GO581539.1 | Avena sativa | cereal | ERKPSEFHPPRARASNMIMKSLALGLLVLACAAVTALGCSGSSRCPGNQCCSKYGYCGLGGDYCGAGCQSGPCYGKRANVDGNIVPGNACSSSSRCPGNQCCSKYGYCGLGGDYCGAGCQSGPCTRAELNENIVPGNACSSSSLCPSNQCCSKWGYCGLGGDYCGSGCQSGPCTGGKLNEDVVSNACSSSSPCPGNQCCSKWGYCGLGGDYCGAGCQSGPCTKAALLSDEMW | 4 |
| GO581912.1 | Avena sativa | cereal | KRKSSEIHPLQVRASNMAMKALALGLLVLACATTTALSCSGGSPCPGNQCCSKYGYCGLGGDYCGAGCQSGPCYGKRANVDGNVIPGNACSSSSPCSGNQCCSKWGYCGLGGDYCGDGCQSGPCTGAKINQDDVPGNACSSSSPCPGNQCCSKWGYCGLGGDYCGSGCQSGPCTGAKLNEDVVPNACSSSSPCSGNQCCSKWGYCGLGGDYCGAGCQSGPCTGAA | 4 |
| GO582252.1 | Avena sativa | cereal | ERKPSEFHPPRARASNMIMKSLALGLLVLACAAVTALGCSGSSRCPGNQCCSKYGYCGLGGDYCGAGCQSGPCYGKRANVDGNIVPGNACSSSSRCPGNQCCSKYGYCGLGGDYCGAGCQSGPCTRAELNENIVPGNACSSSSPCPSNQCCSKWGYCGLGGDYCGSGCQSGPCTGGKLNEDVVSNACSSSSPCPGNQCCSKWGYCGLGGDYCGAGCQSGPCTKAALLSD | 4 |
| GO583188.1 | Avena sativa | cereal | KRKSSEIHPLQARASNMAMKALVLGLLVLACATTTALGCSGGSPCPGNQCCSKYGYCGLGGDYCGAGCQSGPCYGKRANVDGNAIPGNACSSSSPCSGNQCCSKWGYCGLGGDYCGDGCQSGPCTGAKLNQDVVPGNACSSSSPCPGNQCCSKWGYCGLGGDYCGSGCQSGPCTGAKLNEDVVPNACSSSSPCSGNQCCSKWGYCGVGGDYCGAGCQSGPCTGAALLSDEMW | 4 |
| GO585827.1 | Avena sativa | cereal | KRKSSEIHPLQVRASNMAMKALASGLLVLACATTTALSCSGGSPCPGNQCCSKYGYCGLGGDYCGAGCQSGPCYGKRANVDGNVIPGNACSSSSPCSGNQCCSKWGYCGLGGDYCGDGCQSGPCTGAKINQDDVPGNACSSSSPCPGNQCCSKWGYCGLGGDYCGSGCQSGPCTGAKLNEDVVPNACSSSSPCSGNQCCSKWGYCGLGGDYCGAGCQSGPCTGAA | 4 |
| HX827102.1 | Brachypodium distachyon | non-cereal | HNYYLQKLASKRKKKQQDKMMKTKVLTLVVLAFAVAGANAQRCGEQGEGMECPNNLCCSQHGYCGLGGDYCGNGCQNGACYTSKRCGTQANGAICPNNHCCSQYGHCGFGQEYCGDGCQSGPCRANIKCGSQAGGMLCANNLCCSQYGYCGLGSEFCGENCQSGACSGSKQCGRQGGGRVCTNNYCCSQYGHCGLG | 4 |

**Supplementary Table S3 (cont’):** Hololectins with Asn/Asp-containing linkers obtained from data-mining. Accession numbers, species name, category (cereal/non-cereal), precursor sequences and no. of chitin-binding domains (CBD) are indicated:

| **ACCESSION NO.** | **SPECIES** | **CATEGORY** | **PRECURSOR SEQUENCE** | **NO. OF CBD** |
| --- | --- | --- | --- | --- |
| HZTS-2101312 | Sesuvium portulacastrum | non-cereal | MAKTKKSSIGSAAMLAATLYMSTVVLHLAALPVLGQGDRVEQPMQLMPLWQKLGGPECGRQGGGKVCPNNRCCSRWGYCGDTDAFCGNGCQSQCDSGNRCGKDFGGRVCPNGECCSRWGYCGGDEMHCGYGCQSQCDYGRRCGKDFDDRLCPNDECCSEHGYCGVTRAHCEKGCQSQCDYGRCGTEFDDKECEEGMCCSERGYCGVTDAHCGTGCQSQCEQQRCGKEFGGRYCPDWECCSEEGYCGVTDDHCGKGCQSQCRCHLGAKALPATVARLLKLV | 5 |
| IGUH_scaffold_2164028 | Leucodon julaceus | non-cereal | ILIASVFAAAAAAQNCGPSYGNQKCAAGKCCSQYGWCDTGAAYCDPATCLKTYSGTGSKCAAPSDPYASTVPNIDVCGKAQGGVSCPGAGKDKYFYRCCSSQGHCGPKNNIQDQAIYCGAGCQAGFGDCSTNRKKPADPTTPPKTANAGDTCGPIVNAKCATGLCCSGSNFCGTGADFCGAANWCQSKWGKCS | 3 |
| IGUH-2164233 | Schwetschkeopsis fabronia | non-cereal | MIAASISLVLLAFASTVAGNCLVGGRCGVGQCCSAYGFCGSGAQFCGAVTVVQPAPVYATRDCRVVGCSAGYCCSSYGYCGTTSEYCGIGAPAPAVGSCRLTGCPAGSCCSSYGYCGSSGAHCGAVTYGNCGYTACGAGLCCTRYGYCGTIGGYCALQKSSQAEAAPLEGEFEGTATYYNETQASSVYSTCGTERARSLNEDTEQIFTAALNKIQFDPYTVDGIPSNNPICEKKAIVKGPAGQVTVRFVDRCADCKQGDIALTEEAFLAVNGELAPGQTKVEWHFV | 4 |
| IRBN-2014956 | Scapania nemorosa | non-cereal | MVFRGTTNGCRTPRWTLVLLFFTASLQVDWARADQGQCVNKTCPDALCCSKEGYCDTGDAYCGFGCQGGPCYVSSPNSSPPDSGGLGECVNNTCSDGLCCSQHGYCGLSDEYCGFGCRGGSCYRSSHGKNLSTGAIFGIVIASVVGGAILVAILFMAWRRTQSYRTGEKAPQEISKPRLSEDPYNRLL | 2 |
| IRBN-2159154 | Scapania nemorosa | non-cereal | MESLGSEKSWRAQVWLLIFAILCLTDWLQVGIATEEGECDENNVCALGLCCSRFGYCGPGEEYCGSGCQGGSCNVSRSSKTGSEPSHQGECITNTCDAGLCCSNFGFCGHTEKYCGSGCQSGPCFSIQSGKGLSSGVKLFGAVVVAGVLGTILVKAWPKAQGRIGTIYEGCKCRS | 2 |
| IRBN-2159389 | Scapania nemorosa | non-cereal | MLLLGTTNGWSGQRWWLSIVLLCSMASMEVGLVRGQGQCQNNICAAGLCCSQYGYCGNDSAHCGPGCLSGPCLLNSPSSSPSNSDDGSCLSSPCASGLCCSQYNHCGSNSSYCGSGCKGGPCLSNSGAEGVRTGSATVGIALTSAVALMVIFLGFQKSSLRFYV | 2 |
| JZ883150.1 | Triticum aestivum | cereal | STYPGRTLAMKGLFLCALALAFAMVTTHAQLQYCEKRCGKQADGMECPNNLCCSKDGYCGLGVDYCSAAAGCQSGACYDNKICGAQAGGALCPNNHCCSSGGRCGYGSEYCSGSRGCQSGPCWADLKCGHLANGKQCPNNLCCSQYGYCGLGPEFCGARCQNGACSTDKPCGNKANGARCTNNYCCSQYGSCGLGKDYCGTGCQSGACYTPSFLANilKCVP | 4 |

**Supplementary Table S3 (cont’):** Hololectins with Asn/Asp-containing linkers obtained from data-mining. Accession numbers, species name, category (cereal/non-cereal), precursor sequences and no. of chitin-binding domains (CBD) are indicated:

| **ACCESSION NO.** | **SPECIES** | **CATEGORY** | **PRECURSOR SEQUENCE** | **NO. OF CBD** |
| --- | --- | --- | --- | --- |
| KAE8786723.1 | Hordeum vulgare | cereal | MMSTKALTLGAVVVLAIAVAGAHAEQCGQQADGMECPNNLCCSAWGYCGMDSNYCGDGCQSGACYKSKRCGAQAGANAVTCPNNHCCSGDGYCGFGQEYCGDGCQNGPCRANIKCNADKPCLSNFCCSRYGYCGLGVQFCGKGCQSGACHDSVGAAALPLSSIVHE | 3 |
| KAE8816329.1 | Hordeum vulgare | cereal | MMSTRALALGAAAVLAFAAATAHAQRCGEQGSNMECPNNLCCSQYGYCGMGGDYCGKGCQNGACYTSKRCGTQAGGKTCPNNHCCSQWGYCGFGAEYCGAGCQGGPCRADIKCGSQAGGKLCPNNLCCSQWGYCGLGSEFCGEGCQGGACSTDKPCGKAAGGKVCTNNYCCSKWGSCGIGPGYCGAGCQSGGCDGVFAEAIAANSTLVAE | 4 |
| KAF6985492.1 | Triticum aestivum | cereal | MMSTRALALGVSVVLAFTVTTHAQRCGEQGSGMECPNNLCCSQYGYCGMGGDYCGNGCQNGACYTSKRCGSQAGGAVCPNNHCCSQYGHCGFGSEYCGAGCQGGPCHADIKCGSQAGGKLCPNNLCCSQWGYCGLGSEFCGVGCQNGACSTDKPCGKNAGGRACTNNYCCSQWGSCGIGPAYCGAGCQSGGCGAVFAEAIAANSTSTLLKE | 4 |
| KAF6990991.1 | Triticum aestivum | cereal | MMNTRALALGAAVVLAFAAATAHAQRCGEQGSGLECPNNLCCSQYGYCGMGGDYCGNGCQNGACYTSKRCGAQAAGAVCPNNHCCSQYGHCGFGSEYCGAGCQGGPCRADIKCGSQAGGKLCPNNLCCSQWGFCGLGSEFCGVGCQSGACSTDKPCGKNAGGRACTNNYCCSQWGSCGIGPAYCGAGCQSGGCGSVFEAIAANSTSTLLKE | 4 |
| KAF6996730.1 | Triticum aestivum | cereal | MSTRALALGAAVVLAFAAATAHAQRCGEQGSGMECPNNLCCSQYGYCGMGGDYCGNGCQNGACYTSKRCGTQAAGATCPNNHCCSQYGHCGFGEEYCGAGCQGGPCRANIKCGSQAGGKLCPNNLCCSQWGYCGLGSEFCSNGCQSGACSSSKPCGKDNGGRVCTNNYCCSQWGHCGIGPGYCGAGCQSGGCDAVFADAITANSTLLRE | 4 |
| KAF7039008.1 | Triticum aestivum | cereal | MSTKALTLGAAVVLAIAVAGAHAEQCGHAADGMECPNNLCCSAWGYCGMDANYCGDGCQSGACYEPKRCGAQAGGNAVTCPNNHCCSGHGFCGYGQEYCGAGCQNGPCRANIKCSADKPCLSNFCCSQYGYCGLGVEFCGQGCQSGACHDAVGAAALPLSSIVQG | 3 |
| KAF7098018.1 | Triticum aestivum | cereal | MKGLLLCALALAFAVVTTDAQRCGKQGDGMECPNNLCCNKDGYCGLGVTYCNAGAGCQSGACYDNKICGAQAGGALCPSNHCCSSGGRCGYGREYCSNDCQSGPCWDLKCGHLANGRPCPNNLCCSPNGTCGLGPEYCGAGCQNGACSTDKPCGNKANGAPCNNNYCCSQYGSCGLGQDYCGAGCQNGSCN | 4 |

**Supplementary Table S3 (cont’):** Hololectins with Asn/Asp-containing linkers obtained from data-mining. Accession numbers, species name, category (cereal/non-cereal), precursor sequences and no. of chitin-binding domains (CBD) are indicated:

| **ACCESSION NO.** | **SPECIES** | **CATEGORY** | **PRECURSOR SEQUENCE** | **NO. OF CBD** |
| --- | --- | --- | --- | --- |
| KAF7103884.1 | Triticum aestivum | cereal | MKGLLLCALALAFAMVTTHAQLQYCEKRCGKQADGMECPNNLCCSKDGYCGLGVNYCSPAAGCQSGACSDNKICGVQAGGALCPNNHCCSSGGRCGYGSEYCSGSRGCQSGPCWADLKCGHLANGKQCPNNLCCSQHGYCGLGPEFCGVRCQNGACSMDRPCGNKANGARCTNNYCCSPNGSCGLGKDYCGTGCQSGACYTPSFLANILKCVP | 4 |
| KAF7110961.1 | Triticum aestivum | cereal | MKGLLLCALALAFAAVTTHAQLQSCPTRCGKQADGIECPNNLCCSKDGYCGLGVAYCSAGAGCQSGACYDNKICGTQANGTLCPNNHCCGLGGRCGYGREYCSNGCQNGLCWADLKCGHLDNGKLCPNNLCCSQYGYCGLGPEFCGAGCQNGACSTDKPCGNKANGAPCTNNYCCSQYGSCGLGKDYCGTGCQNGACN | 4 |
| KAG2565385.1 | Panicum virgatum | non-cereal | ALALVYLAAAADAQAVPTCGFQVGGAVCGDGRCCSRFGYCGSGPAYCGFGCQSNCNEVGEQCGIQAGGATCANNLCCSQFGFCGLGAQYCGVGCQSNCHAVQRCGIQGGGALCANGLCCSQFGFCGLGAKYCGVGCQKAKAKAVPQCGVQAGGAVCPNGLCCSQFGFCGLGAQYCGVGCQSQCSSG | 4 |
| KAG2565388.1 | Panicum virgatum | non-cereal | MAENSPKRIIAIAGLAAKILVLTALALVYLAAAAEAQAVPTCGFQVGGAVCGDGRCCSRFGYCGSGPAYCGFGCQSNCNEGPAPAPTPSLASVELVKVGEQCGIQAGGATCANNLCCSQFGFCRLGAQYCGVGCQSNCHGSPTIVEPVKAVQWCGIQGGGALCANGLCCSQFGFCGLGAKYCGVGCQSHGAPLATEVKAGECGVQAGGAPCHPPNCCSQYGFCGQGPEYCGAGCQSQCPGP | 4 |
| KAG2565389.1 | Panicum virgatum | non-cereal | MIATAGLAAKALALTALALAYLAAAAEAQTPCGGPQCGGPQCGWQAGGSVCGDGRCCSMYGYCGSGPGYCGLNCQSNCHEGLAAATPVKADPQCGVQAGGAVCANGLCCSQFGFCGLGIKYCGVGCQSQCLETKAKAVPQCGVQAGGAVCPNGLCCSQFGFCGLGAQYCGVGCQSQCSSGAPLAAEVKAGECGVQAGGAPCHPPNCCSQYGFCGQGPEYCGAGCQSQCPGP | 4 |
| KQJ87196.1 | Brachypodium distachyon | non-cereal | MPKKVQILLALALALATHADAYYVQPEEGKCSPECGKDKDNMECPNNLCCSAGGLCGLGSAYCGTGCQSGACSGSRRCGWQNNGATCPNNQCCSWDGYCGLGDWFCRNNKCQSGPCHYDIKCGKGTQNPECPNNFCCNKDGRCGLGAKYCNSDAGCQSGACYDDSTYNQLAMAKKVVLLALALALAATTQAIAKSSSHMGKVPCPAECGKDMDNTECPNNLCCSSGGLCGLGSAYCSATAGCQSGACQVSPQCGADQLCPNNQCCNGGQCGLGSLYCGDGCQNGPCVQDLTCSQGKECPNNFCCNKAGKCGLGDRYCSNTTEVACQSGACYDKITTDSQRCGTQNGG | 3 |

**Supplementary Table S3 (cont’):** Hololectins with Asn/Asp-containing linkers obtained from data-mining. Accession numbers, species name, category (cereal/non-cereal), precursor sequences and no. of chitin-binding domains (CBD) are indicated:

| **ACCESSION NO.** | **SPECIES** | **CATEGORY** | **PRECURSOR SEQUENCE** | **NO. OF CBD** |
| --- | --- | --- | --- | --- |
| KRUQ-2107849 | Porella navicularis | non-cereal | MLITATHVSLLLMSLMYSVAGNCLVGGCGAGLCCSAYGYCGTGYCGAAAGTCRNYGCPAGQCCSQYGYCGNTAAYCGGVSYGNCYSTGCGGGSCCSQNGYCGSYGTYCAVAKFLSGKKAASLEGEFQGQATYYNETMAGAQYSTCGTSRARSLNENDEKIYTAALNEVQFDPYTVDGIPSTNPICQKKAIAKGPKGEIVVQFVDRCQECKEGNIA | 3 |
| M0YEA8 | Hordeum vulgare subsp. Vulgare | cereal | MKGLLLCTLALAFAAVTTHAQLQYCDKRCGKQADGMECPNNLCCSKDGYCGLGANYCSAAAGCQSGACYNNKMCGAQAGGALCPNNHCCSSSGRCGYGSEYCSGSRGCQSGPCWADLKCGHLANGKQCPNNLCCSRQGYCGLGPEFCGGACQNGACSTDKPCGKNANGARCTNNYCCSQYGFCGLGKDYCGTGCESGACYTPSFLADILKCLP | 4 |
| M8CGU9 | Aegilops tauschii | non-cereal | MKGLFLCALALAFAMVTTHAQLQYCEKRCGKQADGMECPNNLCCSKDGYCGLGVDYCSAAAGCPSGARHGNKICGAQAGGALCPNNHCCSSGVRCGYGSEYCGGSRGCQSGPCWADLKCGHLANGPEFCGARCQNGACSTDKPCGNKANGARCTNNYCCSQYGSCGLGKDYCGTGCQSGACYTPSFLANilKCVP | 4 |
| MRKX-2002612 | Phytolacca bogotensis | non-cereal | MKTSNSIVVMLVLVLSSLVLLLPVEGQGHEGHGVGEILLMGKLGEPVCGVSASGRVCPNGHCCSEWGYCGTTNEYCGKGCQSQCDYDRCGQEFGGKKCHHDLCCSQYGWCGYSQDAHCGEGCQSQCNFWRCGKDFGGRICTGNLCCSKYGWCGYTEDHCRDGCQSQCIPSSLLPSPLHRTIAIRKLKANLANMLS | 3 |
| OEL16989.1 | Dichanthelium oligosanthes | non-cereal | MKALALAVLALVYAAASAQAQAQCGSQAGGAVCPKNLCCSQYGYCGIGADYCGVGCQSQCSPPGAFGVGVPVAQCGSQAGGAMCPKNLCCSQYGFCGIGAAYCGVGCQSQCSPPDAFGVGVPVAQCGAQAGGVVCSNNLCCSQYGFCGLGAAYCGTGCQSQCSPGGVGP | 3 |
| OPZX-2050165 | Sesuvium verrucosum | non-cereal | MAKTKKSSIGSVAMLASILYYMSTVMLLQLAVVAVSGQGEVAEQVQVKPLSLWHKLTSFPTCGWQVLGRVCPDGRCCSKWGYCGDTEDYCGSGCQSNCDYLERCGKDFGGRLCPNGECCSQHGYCGVTNAHCDTGCQSQCDYGRCGLEFGDKVCDGGKCCSQWGYCGVTDAHCGNGCQSQCDYGRCGVEFGDGLCPNGECCSENGYCGVTKAHCKMGCQSQCEGLSKANDLPHNTNFTATAARLLKLI | 4 |
| PAN47178.1 | Panicum hallii | non-cereal | MQPMMRATFLLAALAVAYAGAGALAGEQCGRQTGGMLCPSNIQDGRCGIGKGYCGEGCQSGACSPNRKCGHLAGGATCDANQCCSQYGYCGFGVEFCGEGCQSGGVPREPDLRQPGRRVNGYCGLGLEYCGDGCQSGACSADWQCGGRADDGAACANNYCCSKYGYCGLGDDFCGAGCQSGACSSGAAVQGLELVVNQTAASSGGEAIAH | 3 |

**Supplementary Table S3 (cont’):** Hololectins with Asn/Asp-containing linkers obtained from data-mining. Accession numbers, species name, category (cereal/non-cereal), precursor sequences and no. of chitin-binding domains (CBD) are indicated:

| **ACCESSION NO.** | **SPECIES** | **CATEGORY** | **PRECURSOR SEQUENCE** | **NO. OF CBD** |
| --- | --- | --- | --- | --- |
| QKQO-2004260 | Pseudotaxiphyllum elegans | non-cereal | MARHGKMLMLIALAVSLLALTLADGEQCGSQADGATCPNNLCCSKWGYCGSTDAYCNPNEGCQSNCWGEVHALDRGLGDPRCGQAGGGDICNFGRCCSIFDFCGSTDAYCGNDCASQCPPRNGGLRASTGKASYYTRYVPSACYGNDESQLPRNRHMAAVSDGHPNLWKNGEGCGKHYRVRCEGNGCRNADAITIKVVDRC | 2 |
| sp\|E1UYT9.1\| | Stellaria media | non-cereal | MLNMKSFALVMLFATLVGVTIASGPNGQCGPGWGGCRGGLCCSQYGYCGSGPKYCAHNTPLSEIEPTDAGRCSGRGTCSGGRCCSKYGYCGTGPAYCGLGMCQGSCLPDMPNHPAQIQARTEAAQAEAQAEAYNQANEAAQVEAYYQATQAQTQAQPQVEPAVTKAP | 2 |
| sp\|E1UYU0.1\| | Stellaria media | non-cereal | MLNMKSFALLMLFATLVGVTIAYDPNGKCGRQYGKCRAGQCCSQYGYCGSGSKYCAHNTPLSEIEPTAAGQCYRGRCSGGLCCSKYGYCGSGPAYCGLGMCQGSCLPDMPNHPAQIQARTEAAQAEAQAEAYNQANEAAQVEAYYQAQTQAQPQVEPAVTKAP | 2 |
| sp\|P15312.1\| | Hordeum vulgare | cereal | MKMMSTRALALGAAAVLAFAAATAHAQRCGEQGSNMECPNNLCCSQYGYCGMGGDYCGKGCQNGACYTSKRCGTQAGGKTCPNNHCCSQWGYCGFGAEYCGAGCQGGPCRADIKCGSQAGGKLCPNNLCCSQWGYCGLGSEFCGEGCQGGACSTDKPCGKAAGGKVCTNNYCCSKWGSCGIGPGYCGAGCQSGGCDGVFAEAIAANSTLVAE | 4 |
| sp\|Q9AVB0.1\| | Phytolacca americana | non-cereal | MKRISNSIVGMLVVVLSVMLLLPVEGHEGHGVVELIMGKLGAPECGREASGKVCPDDLCCSVFGHCGVSVQHCGDGCQSQCVTNWRCGKDFDDRTCPKKLLCCSKDGWCGNTDAHCGEGCQSQCEQYNWRCGVDFGNRTCPNDLCCSVGGWCGTTDDHCGEGCQSQCEQYNWHCGVDFGNRTCPNDLCCSEWGWCGITEGYCGEGCQSQCNHQRCGKDFAGRTCLNDLCCSEWGWCGSSEAHCGQGCQSNCDYNRCGRNFGFRTCPNELCCSSGGWCGSNDAHCGKGCQSQCDYWRCGVDFSGRVCPQGRCCSAWGWCGDTEEYCEEGCQSQCKLSSLPSPLSQILAIRKLNATIPTMAVE | 7 |
| sp\|Q9AYP9.1\| | Phytolacca americana | non-cereal | MKRSNSIAVMLVLVLSSLMLLLPVEGQGHEGHGVGEILLMGKLGAPVCGVRASGRVCPDGYCCSQWGYCGTTEEYCGKGCQSQCDYNRCGKEFGGKECHDELCCSQYGWCGNSDGHCGEGCQSQCSYWRCGKDFGGRLCTEDMCCSQYGWCGLTDDHCEDGCQSQCDLPTLLPSPLRRIIAIRKLKANLANMLS | 3 |
| VAH20950.1 | Triticum turgidum subsp. durum | cereal | MKMMSTRALALGAAAVLAFAAATAQAQRCGEQGSGMECPNNLCCSHQAGGKLCPNNLCCSQWGYCGLGSEFCGEGCQNGACSTDKPCGKDAGGRVCTNNYCCSKWGSCGIGPGYCGAGCQSGGCDGVFAEAIATNSTLLAE | 2 |

**Supplementary Table S3 (cont’):** Hololectins with Asn/Asp-containing linkers obtained from data-mining. Accession numbers, species name, category (cereal/non-cereal), precursor sequences and no. of chitin-binding domains (CBD) are indicated:

| **ACCESSION NO.** | **SPECIES** | **CATEGORY** | **PRECURSOR SEQUENCE** | **NO. OF CBD** |
| --- | --- | --- | --- | --- |
| VAI80044.1 | Triticum turgidum subsp. durum | cereal | MKGLLLCALALAFAVVTTHAQPQYCEKRCGKQADGMECPNNLCCSKDGYCGLGFDYCSAAAGCQSGACYNNKICGAQAGGAFKYGYCGLGPEFCGAGCQNGACSTDKPCGNQANGARCTNNYCCSQYGSCGLGKDYCGTNCQSGACYNDSILANILKCVP | 3 |
| VAI80052.1 | Triticum turgidum subsp. durum | cereal | MKGLLLLCVLALAFAAVTTHAQLQSCPARCGKQADGMECPNNLCCSKDGYCGLGVDYCSAGAGCQSGACYDNKICGTQANGTFRYGYCGLGPEFCGTGCQNGACSTDKPCGNKANGAACTNNYCCSLYGSCGLGKDYCGTGCQSGACN | 3 |
| VAI80054.1 | Triticum turgidum subsp. durum | cereal | MKGLLLLCVLALAFAAVTTHAQLQSCPARCGKQADGMECPNNLCCSKDGYCGLGVDYCSAGAGCQSGACYDNKICGTQANGTLCPNNHCCSSGGRCGYGSEYCNNGCQNGPCWADLKCGHRDNGKLCPNNLCCSRYGYCGLGPEFCGTGCQNGACSTDKPCGNKANGAACTNNYCCSLYGSCGLGKDYCGTGCQSGACTYSSACYP | 4 |
| VAI92115.1 | Triticum turgidum subsp. durum | cereal | MKGLLLCALALAFAMVTTHAQLQYCEKRCGKQADGMECPNNLCCSKDGYCGLGVNYCSPAAGCQSGACSDNKICGAQAGGALCPNNHCCSSGGRCGYGSEYCSGSRGCQSGPCWADLKCGHLANGKQCPNNLCCSQHGYCGLGPEFCGVRCQNGACSMDRPCGNKANGARCTNNYCCSPNGSCGLGKDYCGTGCQSGACYTPSFLANILKCVP | 4 |
| XP_003575656.1 | Brachypodium distachyon | non-cereal | MKTKVLAPLGAAVVLLALVAVQGAQAQKCGKQGNGMECPNNLCCNQYGNCGIGMDYCSNGCQSGACYKSKRCGTQANGTLCPNNHCCSRLGYCGFGSEYCANNCQSGPCRHDVRCGKEAGGKLCDNNLCCSMWGYCGLGSEFCSIDCQSGPCSYEKLCGKQNGGKACTDGYCCSQHGSCGLGMNYCGQGCQSGSCHNTGLDGNIAFIMTNSTTQ | 4 |
| XP_003575658.2 | Brachypodium distachyon | non-cereal | MKKTMNTKGTLLLSAFVLLTFTFATTTTNAQQCGEQGAGMECPNNLCCSQWGYCGLGAAYCDKGCQSGACYNSKRCGTQANGATCPNNHCCSKHGYCGFGQEYCGDGCQSGPCRADMKCGEQASGKVCANNMCCSQWGYCGLGSEFCGGNCQSGACSAEKPCGKQAGGKGCTNDYCCGADGKCGLGGNYCGRGCQNGGCYKSGGLFDDVDAMMSNSSVLLLPGEANME | 4 |
| XP_003575659.3 | Brachypodium distachyon | non-cereal | MAMTTTKLQQVVVLSTLLAAAALAPLTVHGAVDINLGVPCPKQCGREANNTVCGDNHCCSGDGFCGLGGNYCGSGCQSGACFTNNRCSETSPCPNNQCCSVYGYCGFGQDYCGSGCRNGPCRDDHSCEGGKLCPSNLCCRGKDKKCGLGGNYCSINGDQGCLSGACYDQRCSSAKPCSNGYCCSVHGYCGVGRAYCGGDGSLTLLNGLVCVLS | 4 |

**Supplementary Table S3 (cont’):** Hololectins with Asn/Asp-containing linkers obtained from data-mining. Accession numbers, species name, category (cereal/non-cereal), precursor sequences and no. of chitin-binding domains (CBD) are indicated:

| **ACCESSION NO.** | **SPECIES** | **CATEGORY** | **PRECURSOR SEQUENCE** | **NO. OF CBD** |
| --- | --- | --- | --- | --- |
| XP_004972860.1 | Setaria italica | cereal | MKVLVLCVVALAIATMATDAHAQLLNCPNRCGKQGDGMECPNNLCCSKDGYCGIGSLYCGDGCQSGACHTNQPCGAQAGGAVCPGNLCCSRNGRCGFGSEYCGAGCQGGPCRADIKCGRQAGGKECPNNWCCSQYGYCGMGVEYCGVRCQSGPCIADRPCGLNANGAKCTNNYCCSSSWFCGLGKEYCGDGCQGQFGSCYLQAVADALRLCVIP | 4 |
| XP_010237388.1 | Brachypodium distachyon | non-cereal | MAKKVVLLALALALAATTQAIAKSSSHMGKVPCPAECGKDMDNTECPNNLCCSSGGLCGLGSAYCSATAGCQSGACQVSPQCGADQLCPNNQCCNGGQCGLGSLYCGDGCQNGPCVQDLTCSQGKECPNNFCCNKAGKCGLGDRYCSNTTEVACQSGACYDKITTDSQRCGTQNGG | 3 |
| XP_010237389.1 | Brachypodium distachyon | non-cereal | MMNTKALALGVAILALAAAAAQAQKCGKQGNGMECPNNLCCNQYGNCGLGIEYCSNGCQSGACYTSKRCGAQGNGASCPNNHCCSRWGNCGYGSEYCGAGCQGAPCRDDIKCGQLAAGKLCPNNLCCSQHGYCGLGSEYCGDGCQSGACSAEKLCGKQNGNQACGSDYCCSQYGSCGLGMNYCGQGCQSGSCYKTGLDGTIAFIMTNSTTQ | 4 |
| XP_010237391.1 | Brachypodium distachyon | non-cereal | MMKTKVLTLVVLAFAVAGANAQRCGEQGEGMECPNNLCCSQHGYCGLGGDYCGNGCQNGACYTSKRCGTQANGAICPNNHCCSQYGHCGFGQEYCGDGCQSGPCRANIKCGSQAGGKLCANNLCCSQYGYCGLGSEFCGENCQSGACSGSKQCGRQGGGRVCTNNYCCSQYGHCGLGGDYCGTGCQSGSCSSGLDAIAFIMNNSTTE | 4 |
| XP_010238989.1 | Brachypodium distachyon | non-cereal | MKKKVVILLVLALALAFAAPTHAQLQLLPCPPECGKDKGNTECPNNLCCSAGGLCGLGNAYCGAGCQSGACQLTSCGTDRPCHNNQCCKNEKCGLGSKYCGEGCYSGPCIADQKCSKDNKCPNNFCCNNKGFCGLGDRYCKVDAEVGCQSGPCYNIDDVDDGRSFLGSILDCLLP | 3 |
| XP_010238990.1 | Brachypodium distachyon | non-cereal | MPKKVQILLALALALATHADAYYVQPEEGKCSPECGKDKDNMECPNNLCCSAGGLCGLGSAYCGTGCQSGACSGSRRCGWQNNGATCPNNQCCSWDGYCGLGDWFCRNNKCQSGPCHYDIKCGKGTQNPECPNNFCCNKDGRCGLGAKYCNSDAGCQSGACYDDSVTMGTRQSCRNKEGGTGKSCIKKQGCTRP | 3 |
| XP_020166779.1 | Aegilops tauschii subsp. strangulata | non-cereal | MKGLLLCALALAFAAVTTHAQLQSCPTRCGKQADGMECPNNLCCSKDGYCGLGVDYCSTGAGCQSGACYDNKICGAQANGTLCPNNHCCGSGGRCGHGSEYCSNGCQNGPCWANLKCGHLDNGKLCPNNLCCSQYGYCGLGPEFCGTGCQNGACSTDKPCGNKANGAACTNNYCCSLYGSCGLGKDYCGTGWQSGACN | 4 |

**Supplementary Table S3 (cont’):** Hololectins with Asn/Asp-containing linkers obtained from data-mining. Accession numbers, species name, category (cereal/non-cereal), precursor sequences and no. of chitin-binding domains (CBD) are indicated:

| **ACCESSION NO.** | **SPECIES** | **CATEGORY** | **PRECURSOR SEQUENCE** | **NO. OF CBD** |
| --- | --- | --- | --- | --- |
| XP_020180858.1 | Aegilops tauschii subsp. strangulata | non-cereal | MKGLFLCALALAFAMVTTHAQLQYCEKRCGKQADGMECPNNLCCSKDGYCGLGVDYCSAAAGCQSGACYDNKICGAQAGGALCPNNHCCSSGGRCGYGSEYCSGSRGCQSGPCWADLKCGHLANGKQCPNNLCCSQYGYCGLGPEFCGARCQNGACSTDKPCGNKANGARCTNNYCCSQYGSCGLGKDYCGTGCQSGACYTPSFLANILKCVP | 4 |
| XP_020182158.1 | Aegilops tauschii subsp. strangulata | non-cereal | MKGLLLCALALAFAAVTTHAQLQSCPTRCGKQADGMECPNNLCCSKDGYCGLGVDYCSAGAGCQSGACYDNKICGAQANGTLCPNNHCCSSGGRCGYGREYCSNGCQGGPCWADLKCGHLDNGKLCPNNLCCSQYGYCGLGPEFCGTGCQNGACSTDKPCGNKANGAPCTNNYCCSQYGSCGLGKDYCGTGCQNGACN | 4 |
| XP_020182270.1 | Aegilops tauschii subsp. strangulata | non-cereal | MMSTKALTLGAAVVLAIAVAGAHAEQCGHAADGMECPNNLCCSAWGYCGMDANYCGDGCQSGACYEPKRCGAQAGGNAVTCPNNHCCSGHGFCGYGQEYCGAGCQNGPCRANIKCSADKPCLSNFCCSQYGYCGLGVEFCGQGCQSGACHDAVGAAALPLSSIVQG | 4 |
| XP_020190450.1 | Aegilops tauschii subsp. strangulata | non-cereal | MRKMMSTMALTLGAAVFLAFAAATAQAQRCGEQGSNMECPNNLCCSQYGYCGMGGDYCGKGCQNGACWTSKRCGSQAGGATCPNNHCCSQYGHCGFGAEYCGAGCQGGPCRADIKCGSQSGGKLCPNNLCCSQWGFCGLGSEFCGGGCQSGACSTDKPCGKDAGGRVCTNNYCCSKWGSCGIGPGYCGAGCQSGGCDAVFAGAITANSTLLAE | 4 |
| XP_025791682.1 | Panicum hallii | non-cereal | MQPMMRATFLLAALAVAYAGAGALAGEQCGRQTGGMLCPSNMYCCSQDGRCGIGKGYCGEGCQSGACSPNRKCGHLAGGATCDANQCCSQYGYCGFGVEFCGEGCQAGACRANRTCGSQAGGRPCPDNLCCSVNGYCGLGLEYCGDGCQSGACSADWQCGGRADDGAACANNYCCSKYGYCGLGDDFCGAGCQSGACSSGAAVQGLELVVNQTAASSGGEAIAH | 4 |
| XP_037407679.1 | Triticum dicoccoides | non-cereal | MMSTKALTLGAVVVLAIAMAGAHAEQCGHAADGMECPNNLCCSAWGYCGMDANYCGDGCQSGACYEPKRCGAQAEGNAVTCPNNHCCSGNGYCGYGQEYCGDGCQNGPCRANIKCSADKPCLSNFCCSKYGYCGLGVEFCGQGCQSGACHDAVGAAALPLSSIVQG | 3 |
| XP_037441303.1 | Triticum dicoccoides | non-cereal | MMNTRALALGAAVVLAFAAATAHAQRCGEQGSGLECPNNLCCSQYGYCGMGGDYCGNGCQNGACYTSKRCGAQAAGAVCPNNHCCSQYGHCGFGSEYCGAGCQGGPCRADIKCGSQAGGKLCPNNLCCSQWGFCGLGSEFCGVGCQSGACSTDKPCGKNAGGRACTNNYCCSQWGSCGIGPAYCGAGCQSGGCGSVFAEAIAANSTSTLLKE | 4 |

**Supplementary Table S3 (cont’):** Hololectins with Asn/Asp-containing linkers obtained from data-mining. Accession numbers, species name, category (cereal/non-cereal), precursor sequences and no. of chitin-binding domains (CBD) are indicated:

| **ACCESSION NO.** | **SPECIES** | **CATEGORY** | **PRECURSOR SEQUENCE** | **NO. OF CBD** |
| --- | --- | --- | --- | --- |
| XP_037449610.1 | Triticum dicoccoides | non-cereal | MMSTRALALGVAVVLAFAVTTHAQRCGEQGSGMECPNNLCCSQYGYCGMGGDYCGNGCQNGACYTSKRCGSQAGGAVCPNNHCCSQYGHCGFGSEYCGAGCQGGPCRADIKCGSQAGGKLCSNNLCCSQWGYCGLGSEFCGVGCQNGACSTDKPCGKNAGGRACTNNYCCSQWGSCGIGPAYCGAGCQSGGCGAVFAEAIAANSTSTLLKE | 4 |
| XP_037456830.1 | Triticum dicoccoides | non-cereal | MKGLLLLCVLALAFAAVTTHAQLQSCPARCGKQADGMECPNNLCCSKDGYCGLGVDYCSAGAGCQSGACYDNKICGTQANGTLCPNNHCCSSGGRCGYGSEYCNNGCQNGPCWADLKCGHRDNGKLCPNNLCCSRYGYCGLGPEFCGTGCQNGACSTDKPCGNKANGAACTNNYCCSLYGSCGLGKDYCGTGCQSGACN | 4 |
| XP_037456831.1 | Triticum dicoccoides | non-cereal | MKGLLLCALALAFAVVTTDAQRCGKQGDGMECPNNLCCNKDGYCGLGVTYCNAGAGCQSGACYDNKICGAQAGGALCPSNHCCSSGGRCGYGRKYCSNNCQSGPCWDLKCGHLANGRPCPNNLCCSPNGTCGLGPEYCGAGCQNGACSTDKPCGNKANGAPCSNNYCCSQYGSCGLGQDYCGAGCQNGACN | 3 |
| XP_037467627.1 | Triticum dicoccoides | non-cereal | MKGLLLCALALAFAMVTTHAQLQYCEKRCGKQADGMECPNNLCCSKDGYCGLGVNYCSPAAGCQSGACSDNKICGAQAGGALCPNNHCCSSGGRCGYGSEYCSGSRGCQSGPCWADLKCGHLANGKQCPNNLCCSQHGYCGLGPEFCGVRCQNGACSMDRPCGNKANGARCTNNYCCSQNGSCGLGKDYCGTGCQSGACYTPSFLANILKCVP | 4 |
| XP_037472823.1 | Triticum dicoccoides | non-cereal | MKMMSTRALALGAAAVLAFAAATAQAQRCGEQGSGMECPNNLCCSQYGYCGMGGDYCGKGCQNGACWTSKRCGSQAGGKTCPNNHCCSQYGHCGFGAEYCGAGCQGGPCRADIKCGSQAGGKLCPNNLCCSQWGYCGLGSEFCGEGCQNGACSTDKPCGKDAGGRVCTNNYCCSKWGSCGIGPGYCGAGCQSGGCDGVFAEAIATNSTLLAE | 4 |
| XP_039771702.1 | Panicum virgatum | non-cereal | MKMFMTGRLILLLATLALAGAAVAEAAECGNEAGGMLCPSNLCCSSSGYCGLGRQHCSDGCQSGACYPNKRCGAAAGGATCDANQCCSSLGYCGFGVYYCGDGCQSGACRADAECGGGKVCGDNLCCSSWGYCGLGPEFCGTGCQTGACYDMPSASRAARLAEAAIILSS | 3 |
| XP_039818488.1 | Panicum virgatum | non-cereal | MAENSPKCMIATAGLAAKALALTALALAYLAAAAEAQTPCGGPQCGGPQCGWQAGGSVCGDGRCCSMYGYCGSGPGYCGLNCQSNCHEGLAAATPVKADPQCGVQAGGAVCANGLCCSQFGFCGLGIKYCGVGCQKTKAKAVPQCGVQAGGAVCPNGLCCSQFGFCGLGAQYCGVGCQSQCSSGAPLAAEVKAGECGVQAGGAPCHPPNCCSQYGFCGQGPEYCGAGCQSQCPGP | 4 |

**Supplementary Table S3 (cont’):** Hololectins with Asn/Asp-containing linkers obtained from data-mining. Accession numbers, species name, category (cereal/non-cereal), precursor sequences and no. of chitin-binding domains (CBD) are indicated:

| **ACCESSION NO.** | **SPECIES** | **CATEGORY** | **PRECURSOR SEQUENCE** | **NO. OF CBD** |
| --- | --- | --- | --- | --- |
| Q0JF21 | Oryza sativa | cereal | MTMTSTTTKAMAMAAAVLAAAAVAATNAQTCGKQNDGMICPHNLCCSQFGYCGLGRDYCGTGCQSGACCSSQRCGSQGGGATCSNNQCCSQYGYCGFGSEYCGSGCQNGPCRADIKCGRNANGELCPNNMCCSQWGYCGLGSEFCGNGCQSGACCPEKRCGKQAGGDKCPNNFCCSAGGYCGLGGNYCGSGCQSGGCYKGGDGMAAILANNQSVSFEGIIESVAELV | 4 |
| A0A0E0KMR3 | Oryza punctata | cereal | MTKATVMAVVAVVVSLAALTTTHAQTCGKQNDGMICPNNLCCSQWGYCGLGSDYCGTGCQSGACCSSQRCGSQGGGATCPNNQCCSQYGYCGFGSEYCGTGCQNGPCRADIKCGHNANGKLCPNNMCCSQWGYCGLGSEFCGSGCQSGACCPEKRCGRQAGGAKCPNDYCCSQYGYCGLGGNYCGSGCQSGGCYKGVNGMPAAILSNNQSVSFEGIIKSVAELE | 4 |
| A0A0E0P5D6 | Oryza rufipogon | cereal | MTMTSTTTKAMAMAAAVLAAAAVAATNAQTCGKQNDGMICPHNLCCSQFGYCGLGRDYCGTGCQSGACCSSQRCGSQGGGATCSNNQCCSQYGYCGFGSEYCGSGCQNGPCRADIKCGHNANGELCPNNLCCSQWGYCGLGSEFCGNGCQSGACCPEKRCGKQAGGDKCPNNFCCSAGGYCGLGGNYCGSGCQSGGCYKGGMAAILSSNQSVSFEGIIESVAELV | 4 |

**Supplementary Table S4:** Hololectins without Asn/Asp-containing linkers obtained from data-mining. Accession numbers, species name, category (cereal/non-cereal), precursor sequences and no. of chitin-binding domains (CBD) are indicated:

| **ACCESSION NO.** | **SPECIES** | **CATEGORY** | **PRECURSOR SEQUENCE** | **NO. OF CBD** |
| --- | --- | --- | --- | --- |
| BG368849.1 | Hordeum vulgare | cereal | TTSTKKTRRMKMMSTRALALGAAAVLAFAAATAHAQRCGEQGSNMECPNNLCCSQYGYCGMGGDYCGKGCQNGACYTSKRCGTQAGGKTCPNNHCCSQWGYCGFGARVLRRRLPGRPLPRRHQVRQPGRRQALPQQPLLQPVGLLRPRLRVLR | 2 |
| DN141224.1 | Panicum virgatum | non-cereal | MRSAVLAMKALVLSALLLTFAGVITHAQQCGSQAGGKKCPNNLCCSPWGYCGSGPDYCGNGCQSGPCSGFGTLSAEQCGRQAGNKNCPNNLCCSQWGFCGLGGDYCGNGCQSGPCSGELGAEQ | 2 |
| ERXG-2060416 | Eschscholzia californica | non-cereal | MRMSSSSTSFFLLFLFISSSSSIPFNEWTSDRCGTFGGSVCPEGSCCSIWGYCGNTNDYCVYNCYSQCTKLIPEGRCGTEFSNALCPEGLCCSQWGYCGNTADHCGSGCQSQCSIRCGNVFGDSRCPEGLCCSLWGYCGNTIEHCGAGCQSQCS | 3 |
| FE621365.1 | Panicum virgatum | non-cereal | MRSAVLAMKALVLSALLLTFAGVITHAQQCGSQAGGKKCPNNLCCSPWGYCGTGPDYCGNGCQSGPCSGFGTLSAEQCGRQAGNKNCPNNLCCSQWGFCGLGGDYCGNGCQSGPCSGELGVEQCGRSIDPAPPILIPRHRSGLIHGVEEDPEGVEGPAKGPAHVLQRRSCW | 2 |
| gnl\|onekp\|ABEH_scaffold_2011927 | Heliotropium greggii | non-cereal | SCLVCLLLAATSWADDPLAGRCGPMFGNRPCNGNDCCSIFGWCGRTINHCRPGRCQSQCPQCGTLTGGVRCPEGFCCSKWGFCGQSDDYCAPGNCQSQCT | 2 |
| gnl\|onekp\|HRWG_scaffold_2003950 | Buxbaumia aphylla | non-cereal | MASRGILLALAVAACMLVCAAAAQDQPECGKQAYDAQCLFGKCCSKDGFCGDNDAYCGTGCQNGPCKLMEEVECGVHAYGAECLPRGTCCSASGRCGTGDGYCDQGCQSGDC | 2 |
| gnl\|onekp\|JPYU_scaffold_2004505 | Marchantia polymorpha | non-cereal | SVGLAVVWIVLAASLKLGRAQGGCGSTADNATCANSLCCSSANFCGSTAEYCGTGCQASYGVCGVSPESSPAQGSCEVDRPCQEGYCCSSAGFCGTTTDYCGAGCQSAYGKC | 2 |
| gnl\|onekp\|KVAY_scaffold_2054657 | Tribulus eichlerianus | non-cereal | AFAISCLVCLLLAATSWAADDEGRCGSDFGDKPCNAGACCSKFGWCGTTPSHCAPGNCESQCPQCGPQTGGVLCAEGLCCSKWGFCGNTDAYCGDNCLSQC | 2 |
| gnl\|onekp\|RPQV_scaffold_2032751 | Phymatodocis nordstedtiana | non-cereal | ILGAAVLLLCLLNRATVSAQQCSAIVTCANNLCCSQWGYCGSTSSYCGTRCLSGPCTTPPKYTCGVQAGGQACPSGLCCSQYGSCGFTIGECGAGCQSGPCTSTQFQCGSQSGGLLCSSGLCCSLYGYCGTSSAYCGSGCQSGPCT | 3 |
| gnl\|onekp\|YOXI_scaffold_2001021 | Cylindrocystis brebissonii | non-cereal | IMAPYHRLLISLSFVAASFLLYTSPVVADNCGSGENCGSLCCSQFGFCGSTSDYCGTGCNPNGGTCPSAGGCGTGPNCGTQCCSEFGYCGTTPDYCGTGCNFEGGTCSESSPTPT | 2 |

**Supplementary Table S4 (cont’):** Hololectins without Asn/Asp-containing linkers obtained from data-mining. Accession numbers, species name, category (cereal/non-cereal), precursor sequences and no. of chitin-binding domains (CBD) are indicated:

| **ACCESSION NO.** | **SPECIES** | **CATEGORY** | **PRECURSOR SEQUENCE** | **NO. OF CBD** |
| --- | --- | --- | --- | --- |
| HPXA-2008397 | Ptilidium pulcherrimum | non-cereal | MMIAATGVSLLLLSLVYNVAGNCLISGCGGGGCCSAYGYCGTGYCGAAAGTCLNYGCPAGQCCSQYGYCGNTATYCGGTSYGSCYSTGCGGGLCCSQYGYCGSYGAYGSYCAVAKFLSRKQPVSLEGEFQGQATYYNETMAGADYSTCGTSRARSLDENDEK | 3 |
| RKGT-2057060 | Eschscholzia californica | non-cereal | MRMSSSSTSFFLLFLFISSSSSIPFNEWTSDRCGTFGGSVCPEGSCCSIWGYCGNTNDYCLYNCYSQCTKLIPEGRCGTEFSNALCPEGLCCSQWGYCGNTADHCGSGCQSQCSIRCGNVFGDSRCPEGLCCSLWGYCGNTIEHCGADCQSQCS | 3 |
| sp\|Q9S8M0.2\| | Solanum tuberosum | non-cereal | MKETAISVLALLTLFLLEVVSANELSLPFHLPINETIGLEVFQGINNASPPSPSPLPYPQCGMKKGGGKCIKTGECCSIWGWCGTTNAYCSPGYCQKQCPGPYPEGRCGWQANGKSCPTGTGQCCSNGGWCGTTSDYCASKNCQSQCKLPSPPPPPPPPSPPPPSPPSPPPPSPPPPPPPSPPPPSPPPPSPSPPPPPASPPPPPPALPYPQCGIKKGGGKCIKTGECCSIWGWCGTTNAYCSPGYCQKQCPGPYPEGRCGWQANGKSCPTGTGHCCSNAGWCGTTSDYCAPVNCQAQCNTTTLTSPIKNRMRGIESFMLNVV | 4 |
| XM_021862261.1 | Chenopodium quinoa | cereal | MMKMVKSLVILMVIIMSMHVGGYVMAAGECVRGRCPGGLCCSKFGFCGSGPAYCGGAAEQAEAHPASVAA GECVRGRCPGGLCCSKFGFCGSGPAYCGGAAEQAEAHPATDQVFETTKIPSAADKPASP | 2 |
| XP_039805700.1 | Panicum virgatum | non-cereal | MRSAVLAMKALVLSALLLTFAGVITHAQQCGSQAGGKKCPNNLCCSPWGYCGTGPDYCGNGCQSGPCSGFGTLSAEQCGRQAGNKNCPNNLCCSQWGFCGLGGDYCGNGCQSGPCYGELGAEQCGRQAGGKKCPNSLCCSRFGYCGSGGDYCRNGCQSGPCHTTAGAVPKLSRAFLDQV | 2 |
| XP_039818478.1 | Panicum virgatum | non-cereal | MAENSPKRIIATAGLAAKIMVLTALDHVYLAAAAEAQAVPTCGFQVGGAVCGDGRCCSRFGYCGSGPAYCGFGCQSNCHEGPAPAPTPTLAGVELVKVGEQCGIQAGGAMCANNLCCSQFGFCGLGAKYCGVGCQSQCSGP | 2 |
| XP_039818480.1 | Panicum virgatum | non-cereal | MAENSPKRIIATAGLAAKILVLTALALVYLAAAAEAQAVPTCGFQVGGAVCGDGRCCSRFGYCGSGPAYCGFGCQSNCHEGPAPAPTPTLAGVELVKVGEQCGIQAGGAMCANNLCCSQFGFCGLGAKYCGVGCQSQCSGP | 2 |
| XP_039818483.1 | Panicum virgatum | non-cereal | MAENSPKRIIATAGLAAKILVLTALALVYLAAAAEAQAVPTCGIQVGGAVCGDGRCCSRFGYCGSGPAYCGFGCQSNCHEGPAPAPTPTLAGVELVKVGEQCGIQAGGAMCANNLCCSQFGFCGLGAQYCGVGCQSNCHGSPTTVEPVKAVQRCGIQGGGALCANGLCCSQFGFCGLGAKYCGIGCQSQCLGP | 3 |

**Supplementary Table S4 (cont’):** Hololectins without Asn/Asp-containing linkers obtained from data-mining. Accession numbers, species name, category (cereal/non-cereal), precursor sequences and no. of chitin-binding domains (CBD) are indicated:

| **ACCESSION NO.** | **SPECIES** | **CATEGORY** | **PRECURSOR SEQUENCE** | **NO. OF CBD** |
| --- | --- | --- | --- | --- |
| XP_039833443.1 | Panicum virgatum | non-cereal | MAENSPKRIIATTGLAAKILVLTALALVYLAAAAEAQAVPTCGFQVGGAVCGDGRCCSRFGYCGSGPAYCGFGCQSNCHEGPAPAPTPTLAGVELGKVGEQCGIQAGGAMCANNLCCSQFGFCGLGAKYCGVGCQSQCSGP | 2 |

**Supplementary Table S5:** Hololectins with Asn/Asp-containing linkers obtained from data-mining. Accession numbers, species name, category (cereal/non-cereal) and linker sequences are indicated. Asn (N) and Asp (D) residues in the Asx-containing linkers are shown in bold and underlined:

| **ACCESSION NO.** | **SPECIES** | **CATEGORY** | **LINKER SEQUENCES WITH Asx** | **LINKER SEQUENCES WITHOUT Asx** |
| --- | --- | --- | --- | --- |
| A0A077RWZ6 | Triticum aestivum | cereal | RA**N**IK | YEPKR |
| A0A2G2V3P5 | Capsicum baccatum | non-cereal | GVLETRQSQWEEPAET**D**L |  |
| A0A4S8J4P4 | Musa balbisiana | non-cereal | **ND**SGYQ, **D**GGSGSG**D**S**D**AQ |  |
| ABE77384.1 | Triticum aestivum | cereal | Y**DN**KI, WA**D**LK, ST**D**KP |  |
| ABIJ-2008792 | Selaginella lepidophylla | non-cereal | **N**QQM |  |
| P10968_AGI1_WHEAT | Triticum aestivum | cereal | RA**D**IK, ST**D**KP | WTSKR |
| P02876_AGI2_WHEAT | Triticum aestivum | cereal | RA**D**IK, ST**D**KP | WTSKR |
| AL820037.1 | Triticum aestivum | cereal | RA**D**IK, ST**D**KP | WTSKR |
| BAA02709.1_synthetic_construct | Triticum aestivum | cereal | RA**D**IK, ST**D**KP | WTSKR |
| BG365763.1 | Hordeum vulgare subsp. Vulgare | cereal | RA**D**IK, ST**D**KP | YTSKR |
| BQ246423.1 | Triticum aestivum | cereal | RA**D**IK, IT**D**KP | WTSKR |
| CD901987.1 | Triticum aestivum | cereal | RA**D**IK, ST**D**KP | WTSKR |
| CHJJ-2124255 | Lejeuneaceae sp | non-cereal | GAVTVVQPAPVYATR**D** |  |
| CJ776108.1 | Triticum aestivum | cereal | RA**D**IK, ST**D**KP | YTSKR |
| CJ777748.1 | Triticum aestivum | cereal | RA**N**IK | YTSKR, SSSKP |
| CK169134.1 | Triticum aestivum | cereal | Y**DN**KI, WA**D**LK, ST**D**KP |  |
| EG378972.1 | Leymus cinereus | non-cereal | RA**N**IK, ST**D**KP | YTSKR |
| EG395772.1 | Leymus cinereus | non-cereal | RA**N**IK, ST**D**KP | YTSKR |
| EG396761.1 | Leymus cinereus | non-cereal | RA**N**IK, ST**D**KP | YTSKR |
| EMS45413.1 | Triticum urartu | non-cereal | Y**NN**KI, ST**D**KP |  |
| EMS53669.1 | Triticum urartu | non-cereal | RA**N**IK | YEPKR |
| EMS64751.1 | Triticum urartu | non-cereal | ST**D**KP | YTSKR |
| EMS64753.1 | Triticum urartu | non-cereal | ST**D**KP |  |
| FF351450.1 | Pseudoroegneria spicata | non-cereal | RA**D**IK, ST**D**KP | YTSKR |

**Supplementary Table S5 (cont’):** Hololectins with Asn/Asp-containing linkers obtained from data-mining. Accession numbers, species name, category (cereal/non-cereal) and linker sequences are indicated. Asn (N) and Asp (D) residues in the Asx-containing linkers are shown in bold and underlined:

| **ACCESSION NO.** | **SPECIES** | **CATEGORY** | **LINKER SEQUENCES WITH Asx** | **LINKER SEQUENCES WITHOUT Asx** |
| --- | --- | --- | --- | --- |
| FF356830.1 | Pseudoroegneria spicata | non-cereal | RA**D**IK, ST**D**KP | YTSKR |
| FF367588.1 | Pseudoroegneria spicata | non-cereal | RA**D**IK, ST**D**KP | YTSKR |
| gnl\|onekp\|CMEQ_scaffold_2080348 | Orthotrichum lyellii | non-cereal | EG**D**TRTGPTSAPGATTPM |  |
| gnl\|onekp\|IGUH_scaffold_2164028 | Schwetschkeopsis fabronia | non-cereal | AAPS**D**PYASTVP**N**I**D**V, ST**N**RKKPA**D**PTTPPKTA**N**AG**D**T |  |
| gnl\|onekp\|JADL_scaffold_2042368 | Rhynchostegium serrulatum | non-cereal | **D**KGTVAASAE, **N**KGRTATTGAE |  |
| gnl\|onekp\|LNSF_scaffold_2010351 | Hypnum subimponens | non-cereal | **N**VAEGTTV**D**AASAAE, **N**KGSTYTATT**D**AE |  |
| gnl\|onekp\|QWFV_scaffold_2016436 | Bambusina borreri | non-cereal | SAPSPESGQSPPTPSS**D**GE | SAPGPQAPPPLISSGT |
| gnl\|onekp\|TAVP_scaffold_2078043 | Calliergon cordifolium | non-cereal | **N**KGSTAPM**D**AE |  |
| gnl\|onekp\|YNFJ_scaffold_2022557 | Microtea debilis | non-cereal | G**D**SR |  |
| gnl\|onekp\|YNFJ_scaffold_2173610 | Microtea debilis | non-cereal | GS**N**R |  |
| GO581539.1 | Avena sativa | cereal | YGKRA**N**V**D**G**N**IVPG**N**A, TRAEL**N**E**N**IVPG**N**A, TGGKL**N**E**D**VVS**N**A |  |
| GO581912.1 | Avena sativa | cereal | YGKRA**N**V**D**G**N**VIPG**N**A, TGAKI**N**Q**DD**VPG**N**A, TGAKL**N**E**D**VVP**N**A |  |
| GO582252.1 | Avena sativa | cereal | YGKRA**N**V**D**G**N**IVPG**N**A, TRAEL**N**E**N**IVPG**N**A, TGGKL**N**E**D**VVS**N**A |  |
| GO583188.1 | Avena sativa | cereal | YGKRA**N**V**D**G**N**AIPG**N**A, TGAKL**N**Q**D**VVPG**N**A, TGAKL**N**E**D**VVP**N**A |  |

**Supplementary Table S5 (cont’):** Hololectins with Asn/Asp-containing linkers obtained from data-mining. Accession numbers, species name, category (cereal/non-cereal) and linker sequences are indicated. Asn (N) and Asp (D) residues in the Asx-containing linkers are shown in bold and underlined:

| **ACCESSION NO.** | **SPECIES** | | **CATEGORY** | **LINKER SEQUENCES WITH Asx** | **LINKER SEQUENCES WITHOUT Asx** |
| --- | --- | --- | --- | --- | --- |
| GO585827.1 | Avena sativa | cereal | | YGKRA**N**V**D**G**N**VIPG**N**A, TGAKI**N**Q**DD**VPG**N**A, TGAKL**N**E**D**VVP**N**A |  |
| HX827102.1 | Brachypodium distachyon | non-cereal | | RA**N**IK | YTSKR, SGSKQ |
| HZTS-2101312 | Sesuvium portulacastrum | non-cereal | | **D**SG**N**R, **D**YGRR, **D**YGR | EQQR |
| IGUH_scaffold_2164028 | Leucodon julaceus | non-cereal | | AAPS**D**PYASTVP**N**I**D**V, ST**N**RKKPA**D**PTTPPKTA**N**AG**D**T |  |
| IGUH-2164233 | Schwetschkeopsis fabronia | non-cereal | | GAVTVVQPAPVYATR**D**, GAVTYG**N** | GAPAPAVGS |
| IRBN-2014956 | Scapania nemorosa | non-cereal | | YVSSP**N**SSPP**D**SGGLGE |  |
| IRBN-2159154 | Scapania nemorosa | non-cereal | | **N**VSRSSKTGSEPSHQGE |  |
| IRBN-2159389 | Scapania nemorosa | non-cereal | | LL**N**SPSSSPS**N**S**DD**GS |  |
| JZ883150.1 | Triticum aestivum | cereal | | Y**DN**KI, WA**D**LK, ST**D**KP |  |
| KAE8786723.1 | Hordeum vulgare | cereal | | RA**N**IK | YKSKR |
| KAE8816329.1 | Hordeum vulgare | cereal | | RA**D**IK, ST**D**KP | YTSKR |
| KAF6985492.1 | Triticum aestivum | cereal | | HA**D**IK, ST**D**KP | YTSKR |
| KAF6990991.1 | Triticum aestivum | cereal | | RA**D**IK, ST**D**KP | YTSKR |
| KAF6996730.1 | Triticum aestivum | cereal | | RA**N**IK | YTSKR, SSSKP |
| KAF7039008.1 | Triticum aestivum | cereal | | RA**N**IK | YEPKR |
| KAF7098018.1 | Triticum aestivum | cereal | | Y**DN**KI, W**D**LK, ST**D**KP |  |
| KAF7103884.1 | Triticum aestivum | cereal | | S**DN**KI, WA**D**LK, SM**D**RP |  |
| KAF7110961.1 | Triticum aestivum | cereal | | Y**DN**KI, WA**D**LK, ST**D**KP |  |
| KAG2565385.1 | Panicum virgatum | non-cereal | | **N**EVGEQ | HAVQR, AKAKAVPQ |
| KAG2565388.1 | Panicum virgatum | non-cereal | | **N**EGPAPAPTPSLASVELVKVGEQ | HGSPTIVEPVKAVQW, HGAPLATEVKAGE |
| KAG2565389.1 | Panicum virgatum | non-cereal | | HEGLAAATPVKA**D**PQ | LETKAKAVPQ, SSGAPLAAEVKAGE |
| KQJ87196.1 | Brachypodium distachyon | non-cereal | | HY**D**IK | SGSRR |
| KRUQ-2107849 | Porella navicularis | non-cereal | | GGVSYG**N** | GAAAGT |
| M0YEA8 | Hordeum vulgare subsp. Vulgare | cereal | | Y**NN**KM, WA**D**LK, ST**D**KP |  |

**Supplementary Table S5 (cont’):** Hololectins with Asn/Asp-containing linkers obtained from data-mining. Accession numbers, species name, category (cereal/non-cereal) and linker sequences are indicated. Asn (N) and Asp (D) residues in the Asx-containing linkers are shown in bold and underlined:

| **ACCESSION NO.** | **SPECIES** | **CATEGORY** | **LINKER SEQUENCES WITH Asx** | **LINKER SEQUENCES WITHOUT Asx** |
| --- | --- | --- | --- | --- |
| M8CGU9 | Aegilops tauschii | non-cereal | HG**N**KI, WA**D**LK, ST**D**KP |  |
| MRKX-2002612 | Phytolacca bogotensis | non-cereal | **D**Y**D**R, **N**FWR |  |
| OEL16989.1 | Dichanthelium oligosanthes | non-cereal | SPP**D**AFGVGVPVAQ | SPPGAFGVGVPVAQ |
| OPZX-2050165 | Sesuvium verrucosum | non-cereal | **D**YLER, **D**YGR, **D**YGR |  |
| PAN47178.1 | Panicum hallii | non-cereal | SP**N**RK, SA**D**WQ |  |
| QKQO-2004260 | Pseudotaxiphyllum elegans | non-cereal | WGEVHAL**D**RGLG**D**PR |  |
| sp\|E1UYT9.1\| | Stellaria media | non-cereal | AH**N**TPLSEIEPT**D**AGR |  |
| sp\|E1UYU0.1\| | Stellaria media | non-cereal | AH**N**TPLSEIEPTAAGQ |  |
| sp\|P15312.1\| | Hordeum vulgare | cereal | RA**D**IK, ST**D**KP | YTSKR |
| sp\|Q9AVB0.1\| | Phytolacca americana | non-cereal | VT**N**WR, EQY**N**WR, EQY**N**WH, **N**HQR, **D**Y**N**R, **D**YWR |  |
| sp\|Q9AYP9.1\| | Phytolacca americana | non-cereal | **D**Y**N**R | SYWR |
| VAH20950.1 | Triticum turgidum subsp. durum | cereal | ST**D**KP |  |
| VAI80044.1 | Triticum turgidum subsp. durum | cereal | Y**NN**KI, ST**D**KP |  |
| VAI80052.1 | Triticum turgidum subsp. durum | cereal | Y**DN**KI, ST**D**KP |  |
| VAI80054.1 | Triticum turgidum subsp. durum | cereal | Y**DN**KI, WA**D**LK, ST**D**KP |  |
| VAI92115.1 | Triticum turgidum subsp. durum | cereal | S**DN**KI, WA**D**LK, SM**D**RP |  |
| XP_003575656.1 | Brachypodium distachyon | non-cereal | RH**D**VR | YKSKR, SYEKL |
| XP_003575658.2 | Brachypodium distachyon | non-cereal | Y**N**SKR, RA**D**MK | SAEKP |
| XP_003575659.3 | Brachypodium distachyon | non-cereal | FT**NN**R, R**DD**HS, Y**D**QR |  |
| XP_004972860.1 | Setaria italica | cereal | HT**N**QP, RA**D**IK, IA**D**RP |  |
| XP_010237388.1 | Brachypodium distachyon | non-cereal | VQ**D**LT | QVSPQ |

**Supplementary Table S5 (cont’):** Hololectins with Asn/Asp-containing linkers obtained from data-mining. Accession numbers, species name, category (cereal/non-cereal) and linker sequences are indicated. Asn (N) and Asp (D) residues in the Asx-containing linkers are shown in bold and underlined:

| **ACCESSION NO.** | **SPECIES** | **CATEGORY** | **LINKER SEQUENCES WITH Asx** | **LINKER SEQUENCES WITHOUT Asx** |
| --- | --- | --- | --- | --- |
| XP_010237389.1 | Brachypodium distachyon | non-cereal | R**DD**IK | YTSKR, SAEKL |
| XP_010237391.1 | Brachypodium distachyon | non-cereal | RA**N**IK | YTSKR, SGSKQ |
| XP_010238989.1 | Brachypodium distachyon | non-cereal | IA**D**QK | QLTS |
| XP_010238990.1 | Brachypodium distachyon | non-cereal | HY**D**IK | SGSRR |
| XP_020166779.1 | Aegilops tauschii subsp. strangulata | non-cereal | Y**DN**KI, WA**N**LK, ST**D**KP |  |
| XP_020180858.1 | Aegilops tauschii subsp. strangulata | non-cereal | Y**DN**KI, WA**D**LK, ST**D**KP |  |
| XP_020182158.1 | Aegilops tauschii subsp. strangulata | non-cereal | Y**DN**KI, WA**D**LK, ST**D**KP |  |
| XP_020182270.1 | Aegilops tauschii subsp. strangulata | non-cereal | RA**N**IK, ST**D**KP | YEPKR |
| XP_020190450.1 | Aegilops tauschii subsp. strangulata | non-cereal | RA**D**IK, ST**D**KP | WTSKR |
| XP_025791682.1 | Panicum hallii | non-cereal | SP**N**RK, RA**N**RT, SA**D**WQ |  |
| XP_037407679.1 | Triticum dicoccoides | non-cereal | RA**N**IK | YEPKR |
| XP_037441303.1 | Triticum dicoccoides | non-cereal | RA**D**IK, ST**D**KP | YTSKR |
| XP_037449610.1 | Triticum dicoccoides | non-cereal | RA**D**IK, ST**D**KP | YTSKR |
| XP_037456830.1 | Triticum dicoccoides | non-cereal | Y**DN**KI, WA**D**LK, ST**D**KP |  |
| XP_037456831.1 | Triticum dicoccoides | non-cereal | Y**DN**KI, W**D**LK |  |
| XP_037467627.1 | Triticum dicoccoides | non-cereal | S**DN**KI, WA**D**LK, SM**D**RP |  |
| XP_037472823.1 | Triticum dicoccoides | non-cereal | RA**D**IK, ST**D**KP | WTSKR |
| XP_039771702.1 | Panicum virgatum | non-cereal | YP**N**KR, RA**D**AE |  |
| XP_039818488.1 | Panicum virgatum | non-cereal | HEGLAAATPVKA**D**PQ | QKTKAKAVPQ, SSGAPLAAEVKAGE |
| Q0JF21 | Oryza sativa | cereal | RA**D**IK | CSSQR, CPEKR |
| A0A0E0KMR3 | Oryza punctata | cereal | RA**D**IK | CSSQR, CPEKR |
| A0A0E0P5D6 | Oryza rufipogon | cereal | RA**D**IK | CSSQR, CPEKR |

**Supplementary Table S6:** Hololectins without Asn/Asp-containing linkers obtained from data-mining. Accession numbers, species name, category (cereal/non-cereal) and linker sequences are indicated:

| **ACCESSION NO.** | **SPECIES** | **CATEGORY** | **LINKER SEQUENCES WITHOUT Asx** |
| --- | --- | --- | --- |
| BG368849.1 | Hordeum vulgare | cereal | YTSKR |
| DN141224.1 | Panicum virgatum | non-cereal | SGFGTLSAEQ |
| ERXG-2060416 | Eschscholzia californica | non-cereal | TKLIPEGR, SIR |
| FE621365.1 | Panicum virgatum | non-cereal | SGFGTLSAEQ |
| gnl\|onekp\|ABEH_scaffold_2011927 | Heliotropium greggii | non-cereal | PQ |
| gnl\|onekp\|HRWG_scaffold_2003950 | Buxbaumia aphylla | non-cereal | KLMEEVE |
| gnl\|onekp\|JPYU_scaffold_2004505 | Marchantia polymorpha | non-cereal | GVSPESSPAQGS |
| gnl\|onekp\|KVAY_scaffold_2054657 | Tribulus eichlerianus | non-cereal | PQ |
| gnl\|onekp\|RPQV_scaffold_2032751 | Phymatodocis nordstedtiana | non-cereal | TTPPKYT, TSTQFQ |
| gnl\|onekp\|YOXI_scaffold_2001021 | Cylindrocystis brebissonii | non-cereal | PSAGG |
| HPXA-2008397 | Ptilidium pulcherrimum | non-cereal | GAAAGT, GGTSYGS |
| RKGT-2057060 | Eschscholzia californica | non-cereal | TKLIPEGR, SIR |
| sp\|Q9S8M0.2\| | Solanum tuberosum | non-cereal | PGPYPEGR, KLPSPPPPPPPPSPPPPSPPSPPPPSPPPPPPPSPPPPSPPPPSPSPPPPPASPPPPPPALPYPQ, PGPYPEGR |
| XM_021862261.1 | Chenopodium quinoa | cereal | GAAEQAEAHPASVAAGE |
| XP_039805700.1 | Panicum virgatum | non-cereal | SGFGTLSAEQ |
| XP_039818478.1 | Panicum virgatum | non-cereal | HEGPAPAPTPTLAGVELVKVGEQ |
| XP_039818480.1 | Panicum virgatum | non-cereal | HEGPAPAPTPTLAGVELVKVGEQ |
| XP_039818483.1 | Panicum virgatum | non-cereal | HEGPAPAPTPTLAGVELVKVGEQ, HGSPTTVEPVKAVQR |
| XP_039833443.1 | Panicum virgatum | non-cereal | HEGPAPAPTPTLAGVELGKVGEQ |
